# Stochastic Blockmodeling of the Modules and Core of the *Caenorhabditis elegans* Connectome

**DOI:** 10.1371/journal.pone.0097584

**Published:** 2014-07-02

**Authors:** Dragana M. Pavlovic, Petra E. Vértes, Edward T. Bullmore, William R. Schafer, Thomas E. Nichols

**Affiliations:** 1 Department of Statistics and Warwick Manufacturing Group, University of Warwick, Coventry, United Kingdom; 2 Brain Mapping Unit, Behavioural and Clinical Neuroscience Institute, Department of Psychiatry, University of Cambridge, Cambridge, United Kingdom; 3 GlaxoSmithKline, Clinical Unit Cambridge, Addenbrooke's Hospital, Cambridge, United Kingdom; 4 Cambridgeshire and Peterborough NHS Foundation Trust, Cambridge, United Kingdom; 5 Medical Research Council Laboratory of Molecular Biology, Cell Biology Division, Cambridge, United Kingdom; Georgia State University, United States of America

## Abstract

Recently, there has been much interest in the community structure or mesoscale organization of complex networks. This structure is characterised either as a set of sparsely inter-connected modules or as a highly connected core with a sparsely connected periphery. However, it is often difficult to disambiguate these two types of mesoscale structure or, indeed, to summarise the full network in terms of the relationships between its mesoscale constituents. Here, we estimate a community structure with a stochastic blockmodel approach, the Erdős-Rényi Mixture Model, and compare it to the much more widely used deterministic methods, such as the Louvain and Spectral algorithms. We used the *Caenorhabditis elegans* (*C. elegans*) nervous system (connectome) as a model system in which biological knowledge about each node or neuron can be used to validate the functional relevance of the communities obtained. The deterministic algorithms derived communities with 4–5 modules, defined by sparse inter-connectivity between all modules. In contrast, the stochastic Erdős-Rényi Mixture Model estimated a community with 9 blocks or groups which comprised a similar set of modules but also included a clearly defined core, made of 2 small groups. We show that the “core-in-modules” decomposition of the worm brain network, estimated by the Erdős-Rényi Mixture Model, is more compatible with prior biological knowledge about the *C. elegans* nervous system than the purely modular decomposition defined deterministically. We also show that the blockmodel can be used both to generate stochastic realisations (simulations) of the biological connectome, and to compress network into a small number of super-nodes and their connectivity. We expect that the Erdős-Rényi Mixture Model may be useful for investigating the complex community structures in other (nervous) systems.

## Introduction

The past thirty years have seen increasing interest in the systems-level understanding of structures as diverse as the global economy [1], ecosystems [2], living cells [3], power grids [4] and more. To obtain deeper insights into the operational mechanisms governing these systems, the scientific focus has gradually moved away from the analysis of their isolated components to the ways in which these components interact to perform the functions that characterise the system as a whole. In this manner, a wide range of systems can all be studied as networks, defined through their elements (vertices or nodes) and the connections (edges) that link them. A system's functional properties can then be studied in terms of the connection structure that is associated with its network.

In the context of the brain, the same approach can be used to study how simple elements (e.g., neurons) are organised into circuits to process information. This allows us to gain greater insights than the study of a single, isolated element would normally provide. For example, individual neurons can engage in complex physiological responses that are triggered by interactions between larger numbers of neurons locked in circuits. Thus, the knowledge of such circuits can provide a better understanding of brain activity [5]. In addition, brain and mind disorders are increasingly thought of in terms of damage to the connections between brain regions [6].

Although network analysis has great potential for addressing some of the key questions in neuroscience, its application at a cellular scale is only possible for one complete nervous system, namely that of *Caenorhabditis elegans*. Indeed, *C. elegans* is the only organism whose connectome (or pattern of neuronal connections) has been mapped extensively at the level of neurons and synapses, and it has therefore become a gold standard system for brain connectivity analyses [7]–[9].

However, even this simple model system consists of close to 300 individual neurons and more than 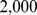 edges, making its analysis non-trivial. Nevertheless, a tractable analysis is possible when it is considered that networks with high functional requirements tend to be organised in terms of homogeneous clusters that define different levels of functional hierarchy [10], [11]. Indeed, decomposing such networks into a collection of functionally coherent clusters, known as mesoscale organisation or community structure, can serve as a meaningful summary of the overall network's structure.

However, one of the underlying difficulties in the inference of a network's community structure is that the concept of “community” is not precisely defined. This degree of arbitrariness has generated diverse methodological solutions which can, in a broad sense, be classified into two groups: deterministic methods based on heuristic objective functions, and model-based methods that relate the observable data to unobservable parameters of interest with a statistical model [12].

Within the framework of deterministic methods, an exhaustive search over the sample space of all possible community structures and their corresponding partitions is astronomically large [13]. Nevertheless, various methods are available for particular definitions of community structure. One class of such methods (i.e., community detection algorithms) searches for groups of nodes, called modules, that comprise a high density of links within them and a lower density of links between them. The popularity of these algorithms stems from the fact that they are computationally feasible and, in particular, some of them can determine an optimal number of modules. We consider two such deterministic methods, namely the Fast Louvain algorithm [14] and the Spectral algorithm [15], [16], chosen because of their widespread use in the literature.

**Figure 1:**
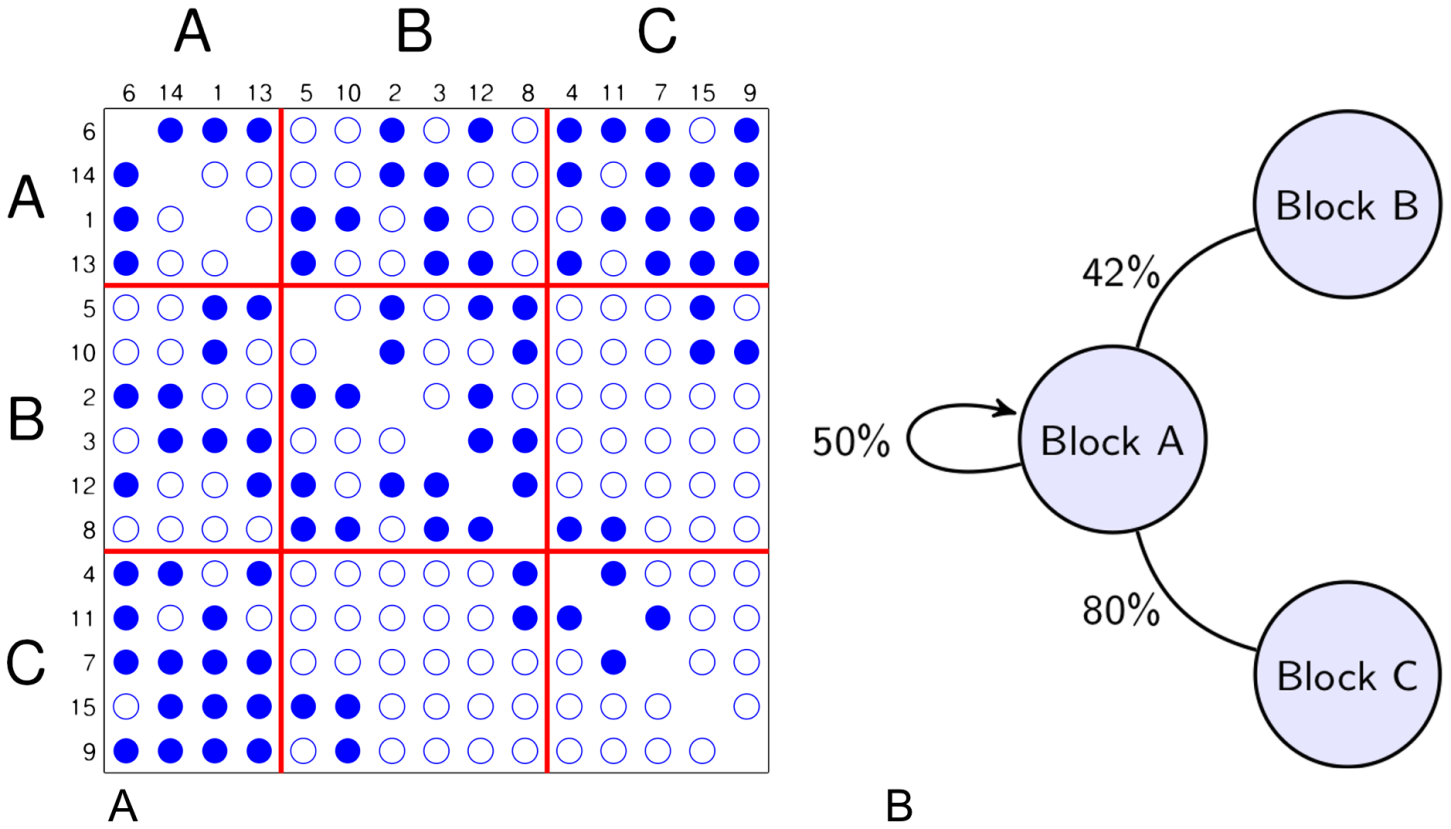
Network decomposition in terms of stochastic equivalence. (**A**) Block decomposition of an undirected network on 15 nodes (numbered from 1 to 15), where the blue circles mark the presence of connection, the empty circles mark the absence of connection and the red lines demarcate Block A, B and C. (**B**) Compressed, stochastic representation of Block A in the network's block decomposition. Block A is defined as a group of stochastically equivalent nodes, each node having a 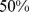 chance to form an edge with another node in Block A, a 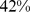 chance to form an edge with another node in block B and a 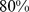 chance to form an edge with another node in Block C.

In contrast to this, model-based methods use statistical tools to estimate the community structure in a process which is generally known as the stochastic blockmodeling. The origins of this approach can be found in the sociometric literature, dating back to the work of Lorrain and White [17] and others [12], [18], [19] who formulated methods similar to those of modern day network compression, as well as other articles that developed stochastic blockmodels [20]–[22]. Although there are various types of stochastic blockmodels which differ in terms of parametrisation and estimation strategies, they share a consistent view of the community structure in a network. In particular, the observed network is seen as a random realisation from a sample space of all possible networks, and community structure is seen as a collection of blocks; all nodes in a given block share the same probabilities of connection with other nodes in the network. Formally, this is known as the stochastic equivalence (see Figure 1). The distinctive feature of this approach is that it groups nodes together according to their similarity of connection patterns, in contrast to other community detection algorithms that groups nodes solely on the basis of high density of connections. With such approach, we can recognise a group of densely connected nodes as comprised of distinct groups, distinguished by their extra-block connections (see Figure 1 (b), where Block A's connections to Block C differentiate it from Block B, despite similar connections within and between Blocks A & B).

Identifying the best possible decomposition of a network into such blocks and identifying the associated connectivity rules is an area of active research in statistics. While the classic stochastic blockmodel proposed by Nowicki and Snijders [23] can handle small networks (i.e., 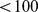 nodes), the more recent Erdős-Rényi Mixture Model (ERMM) proposed by Daudin et al. [24] can handle networks with several thousand nodes. In the ERMM, each block is modelled as a small Erdős-Rényi network with a common probability of internal connections, and the relationship between each block pair is also modelled as a separate Erdős-Rényi network specified by a probability of inter-group connections (as in Figure 1). Fitting the ERMM requires the estimation of the total number of blocks, the connection probabilities within each block and between each block pair, as well as the assignment of nodes into blocks. The output consists of both an estimated community structure and a simple model for the connectivity between blocks.

In this paper, we compare the quality of the community structure identified by the ERMM to those found by more traditional community detection methods. We quantify the results of all 3 methods using prior data on the neurons (nodes) and synapses (edges) of the *C. elegans* connectome. We also illustrate the ability of the ERMM to capture other forms of mesoscale structure in the network beyond the presence of modules and to summarise the structure as a compressed network of super-nodes. In particular, we show that the ERMM provides a natural framework for identifying the core-periphery structure, defined as the densely connected core and sparsely connected periphery [25], a structure that cannot be identified by the deterministic community detection algorithms. Furthermore, we show that the ERMM can isolate biologically coherent groups of neurons and that it also provides a generative model yielding, for example, a good approximation of the network's degree distribution and means to simulate new data.

## Data and Methods

### Data

The neuronal network of the adult nematode *C. elegans* was first described in the publication by White et al. [26] and was recently revised by Chen et al. [27] and Varshney et al. [28]. It expresses the regime of connections between the animal's 282 somatic neurons and classifies them with respect to their type and direction (http://www.wormatlas.org/neuronalwiring.html, accessed 15th June 2013).

In our analysis, we consider a subset of this data where 3 disconnected neurons (VC06, CANL and CANR) are excluded from the set and we take all connections to be undirected. Furthermore, while the connections are distinguished in terms of their type (chemical synapses, gap junctions and neuromuscular junctions), we treat all connections as binary, that is, we assign value 1 if some type of connection exist and 0 otherwise. This yields a 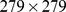 binary and symmetric adjacency matrix with 2287 edges that defines the *C. elegans* network.

For an external evaluation of the community estimates, we use *categorical* and *quantitative* characteristics of the neurons (node-wise features) and *quantitative* characteristics of the edges (edge-wise features), as summarised in Table 1.

**Table 1:** Prior biological features of the *C. elegans* connectome.

Name	Type	Node-wise	Edge-wise
Ganglion Classification (10 groups)	Categorical	√	-
Neuron Type (4 groups)	Categorical	√	-
Neuron Class (103 groups)	Categorical	√	-
Locomotion Circuit (84 nodes)	Categorical	√	-
Anatomical Location	Quantitative	√	-
Anatomical Distance	Quantitative	-	√
Birth Time	Quantitative	√	-
Birth Time Difference	Quantitative	-	√
Lineage Distance	Quantitative	-	√

There is a large body of knowledge on the individual neurons, producing node-wise features. For example, we use the classification of neurons into ten anatomically defined ganglia (“Ganglion classification”), the classification of neurons by their circuitry (“Neuron type”) defined by four groups (sensory, motor, interneurons and polymodal neurons), as well as topological and synaptic division of neurons (“Neuron class”) defined by 103 groups [28], [29]. We also consider ventral nerve cord motor neurons involved in locomotion, egg-laying and possibly avoidance (broadly labelled as “Locomotion circuit” in Table 1) which was described by Haspel et al. [30] using connection data from Chalfie and White [31], Von Stetina et al. [32], Altun and Hall [33], and Chen et al. [27]. Explicitly, this circuit is composed of 84 neurons, of which 74 are motor neurons (excluding VC06) that comprise eight neuron classes. Four of these classes are connected to ventral muscles (VA, VD,VB and VC) while the other four classes are connected to dorsal muscles (AS, DA, DD and DB). The remaining 10 neurons are interneurons (AVA, AVD and AVE; AVB and PVC) promoting backward and forward motion. Although the connection data used in our analysis do not include neuromuscular connections, the circuit presented by Haspel et al. [30] provides some invaluable insights that are beneficial to the evaluation and comparison of the results obtained in our analysis. The remaining set of the node-wise features includes “Anatomical Location” (longitudinal and sectional positions) of the cell body (soma) and the “Birth Time” of each neuron (http://www.biological-networks.org/?page_id=25, accessed 15th June 2013) [34].

Edge-wise features include the “Anatomical Distance” (Euclidean distance between each neuron pair), the “Birth Time Difference” (for each neuron pair, we take an absolute difference in their birth times) and the “Lineage Distance” (for each neuron pair, this is the sum of total divisions to the most recent common ancestor cell) [35].

### Methods

Our analysis consists of two stages. In the first stage, we derive community structures of the *C. elegans* neural network using 3 different methods, as described next. In the second stage, we estimate how well each network decomposition explains the system's known prior biological properties. The general techniques used for this part of the analysis are summarised in Section “Evaluation Methods”.

We first fix our general notation, but emphasise that the terms “network” and “graph” are used interchangeably. A graph 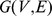 is defined as an object formed by a finite set of vertices (nodes) *V* of size *n* and a list of unordered pairs of vertices *E* (edge list) of size *m*. For a simple graph (i.e., graph without multiple edges or self connected vertices), the adjacency matrix 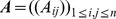 is symmetric and binary, that is, its elements *A_ij_* take value 1 if there is an edge between vertices *V_i_* and *V_j_* and 0 otherwise. The degree of each vertex is 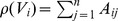, the number of edges connected to a vertex, while the set of all degrees is 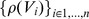. Additionally, a graph can be characterised by a clustering coefficient that measures the tendency of its edges to form clusters. The clustering coefficient, defined by Newman [24], [36] is
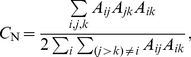
(1)the prevalence of fully connected triplets of nodes among the set of triplets that have at least two connections.

#### The Erdős-Rényi Mixture Model (ERMM)

The Erdős-Rényi (ER) model for a graph [37], [38] specifies that edges occur independently with a common probability. Real world graphs are rarely so homogeneous, and the ER model is generally not useful. In contrast, the Erdős-Rényi Mixture Model [23], [24], [39]–[41] poses an ER model on subsets of edges within the graph.

In the ERMM, the adjacency matrix is treated as a random variable denoted by 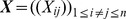 and the nodes are assumed to be allocated into *Q* unknown (latent) groups or blocks, indexed by 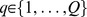. We record the group assignment of each node *V_i_* with a 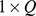 dimensional random (classification) vector 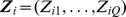, whose elements *Z_iq_* take value 1 if *V_i_* belongs to the *q*-th group and 0 otherwise; 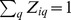 as each node belongs to exactly one group. The set 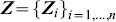 then consists of independent, identically distributed random variables, each following a single trial multinomial distribution
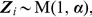
(2)where 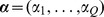 is a 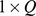 dimensional vector whose elements satisfy the constraint 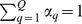. The elements of 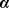 describe the size or prevalence of each group, or, alternatively, can be interpreted as the probability that a randomly chosen node is contained in the *q*-th group. Note that different assumptions about the distribution of 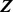 are also possible (see, e.g., the recent publication of Latouche et al. [42] who proposed an overlapping stochastic blockmodel).

The ERMM specifies that, given the group (block) assignments of the vertices, the elements of 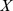 are conditionally independent Bernoulli random variables with rates given by their corresponding elements in the connectivity matrix 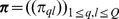. In other words, if a vertex *V_i_* belongs to group *q* and a vertex *V_j_* belongs to group *l*, then
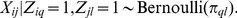
(3)As is often the case with mixture models, the likelihood is stated as an incomplete data problem which is optimised for different values of *Q*, that is, 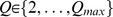. In the ERMM, however, such optimisation is particularly challenging. Nevertheless, the estimating equations of the model's parameters (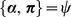) can still be obtained with an approximate *variational method* [43], [44]. With an additional parameter 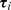
(i.e., the variational parameter for *V_i_*), the estimating equations proposed in [24] are
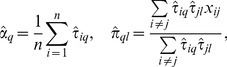
(4)

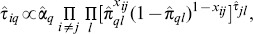
(5)where we employ the usual statistical convention of lower Roman variables, *x_ij_*, to denote the observed version of the random data, *X_ij_*.

For each node, the largest variational parameter estimate 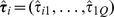 determines the classification vector estimate 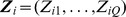


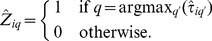
(6)The estimates just described depend on *Q*, the total number of partitions. To compare across different *Q*, the *Integrated Classification Likelihood* (ICL) criterion is used. For a model 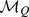 with *Q* groups, the ICL criterion is
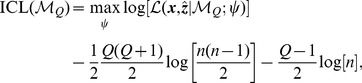
(7)where 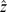 is an estimate of *z* and 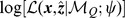
is the complete data log likelihood,
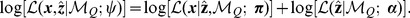
(8)The details of each likelihood term as well as the derivation of the ICL criterion are presented in the Supplementary Text in File SI.

Intuitively, the ICL criterion considers the evidence for the clustered data (i.e., 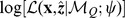), and, at the same time, it uses the term (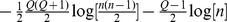) to penalise the model's complexity and, therefore, preserve the simplicity and parsimony of the selected model. Hence, it is generally harder to select a model with a larger number of groups.

Using a Poisson approximation for a binomial distribution, the ERMM models the degree distribution as a mixture of Poisson distributions,
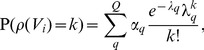
(9)where 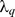 is the Poisson rate for the *q*-th group, 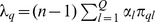.

Finally, Daudin, Picard and Robin in [24] proposed that the fitted ERMM can be used to estimate the Newman clustering coefficients (see Eq. (1)) as
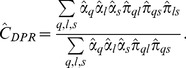
(10)For further mathematical details on the ERMM, see Supplementary Text in File S1.

#### The Spectral and Fast Louvain Algorithms

In contrast to the ERMM, the Spectral and Fast Louvain algorithms are deterministic methods that assess the goodness of a graph partition with an objective function known as *modularity* [45]. Central to the definition of modularity is the difference between the observed edge (*A_ij_*) and the expected number of edges (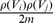) in an equivalent graph with *m* edges and with randomly connected vertices [46]–[48]. Modularity is defined as
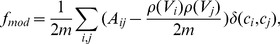
(11)where *c_i_* and *c_j_* represent the groups of vertices *V_i_* and *V_j_*, and 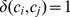 if *V_i_* and *V_j_* are located in the same module and 0 otherwise.

#### The Spectral Algorithm

The Spectral algorithm [15], [49] optimises modularity (Eq. (11)) by utilising the eigenvalues and eigenvectors associated with the modularity matrix 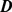 with elements defined as
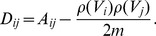
(12)The graph is split into two modules by setting an indicator vector 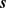 such that 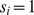 if the vertex *V_i_* is located in the module and 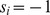 otherwise. Hence, the modularity can be expressed as
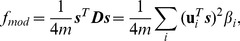
(13)where 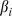 is the eigenvalue of 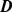 corresponding to the eigenvector 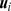. Observe that, for a given 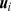 and a 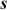 consisting only of 1's or −1's, the inner product vector 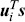 is maximised by 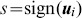. This creates two groups, of not necessarily equal size, and each group is in turn split with the additional contribution to modularity 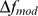 being defined as
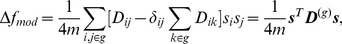
(14)where 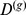 is 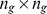 (for a group *g* of size *n_g_*) whose elements are: 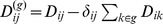. When no more positive eigenvalues are found, the algorithm stops. More details on the Spectral Algorithm can be found in Supplementary Text in File S1.

#### The Fast Louvain Algorithm

The Fast Louvain algorithm [14] optimises modularity (Eq. (11)) in two stages that are repeated iteratively. The algorithm is initialised by assigning each vertex to its own module and, hence, the initial number of groups is equal to the number of vertices. In the first stage, for each vertex *V_i_*, the algorithm considers each of its neighbours and computes the gain of modularity that would have been obtained if the vertex *V_i_* was placed in the same module as its neighbour *V_j_*. The vertex *V_i_* is assigned to the module for which this gain is the largest or, in the case of no positive gain, the vertex stays in its initial module. This process is applied sequentially, cycling through every vertex until no individual move can improve the modularity at which point the first stage stops.

In the second stage, the algorithm builds a new network whose vertices are identified as the modules found in the first stage. This gives a simplified community structure that is used as the initialisation for the next pass of the first stage. These two stages are repeated until the maximal modularity is attained.

#### Practical Aspects

Community estimation methods are notoriously sensitive to the initial starting conditions (see e.g., [50]). Each method begins with some sort of random initialisation that typically will lead to a local optimum of the objective function (i.e., ICL or modularity). Thus, for all three methods considered, we use multiple random restarts of the algorithm and take the solution that provides the greatest value of the objective function.

### Evaluation Methods

To measure the similarity between a partition (i.e., complete segmentation of a graph into a set of groups) and some known biological classifications, we use the Adjusted Rand Index (ARI) [51], [52]. This measure is a modification of the Rand Index (RI) [53], that is expressed as the fraction of vertex pairs that are consistent: a vertex pair is consistent between two partitions if either (a) the vertex pair is within the same group in both partitions, or (b) the vertex pair is split between two groups in both partitions. The interpretation of the RI depends on the number of groups [54], whereas the ARI is adjusted for chance agreement and number of groups [52]. It is defined as
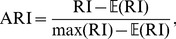
(15)where the expectation is computed assuming a hypergeometric distribution of the counts of consistent vertex pairs. ARI scores range from 0 to 1, and indicate the proportion of overlap; for example, if two partitions have an ARI score of 0.6, this means that 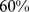 of the nodes are classified in the same groups.

To assess the quality of a partition with respect to quantitative biological features, we use the Intra-class Correlation Coefficient (ICC). The ICC measures the variance that a partition explains in a continuous variable. As per best practice, we estimate the ICC with a mixed effects model [55]. For a node-wise measure, if we denote 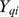 as the measure on the *i*-th neuron in the *q*-th group, the mixed effects model is
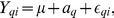
(16)where *a_q_* is the random effect of the *q*-th group, 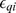 is the random error term and 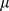 is the population mean. The random terms *a_q_* and 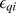 are mutually independent and each are independently and identically distributed normal random variables: 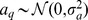
and 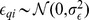. The ICC is defined as the proportion of total variance explained by the between group variance,
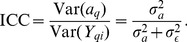
(17)In other words, the ICC tells us how homogeneous the biological feature is within the partitions of a proposed network decomposition. Note that, here, we defined the ICC for node-wise measures (e.g., anatomical location of neuron), but it can be also defined for edge-wise measures (e.g., Euclidean distance between neurons). While edge-wise measures may violate the independence assumption of the mixed effect model, the ICC will still be a useful metric to compare biological validity of different partitions.

Ideally, we would conduct a hypothesis test on the difference in fit between different community estimates. However, because the implied models are not nested, a traditional hypothesis test cannot be employed. Nevertheless, we are able to use model selection metrics, such as the Akaike Information Criterion (AIC) [56]. The AIC can be viewed as a measure of distances between a fitted model (i.e., an estimated partition) and the unknown true model (i.e., the true partition). Denoting 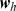 to be the model under consideration (i.e., one of the ERMM, Spectral or Louvain methods), the AIC score is defined as
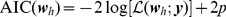
(18)where 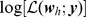 is the log likelihood of the corresponding mixed effect model (Eq. (16)) and *p* is the number of parameters in the model (here, *p* = 3). The preferred model is the one with the smallest AIC score (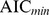). While the AIC is not an absolute measure, the differences in the AIC scores provide a way to compute approximate probabilities. In particular, the relative likelihood of the model 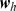 compared to the model that minimises the AIC is given as
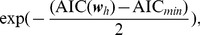
(19)and represents the relative strength of evidence for this model. Equivalently, this tells us how probable it is that the model 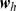 minimises the distance from the true model. As a general rule of guidance, it has been suggested [57] that, if the likelihood value 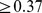
(or, equivalently 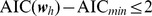), there is a substantial evidence that this model is equally useful; if the value is contained in the interval 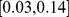 (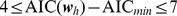), then, there is considerably less evidence; and, finally, for values that are strictly smaller than 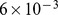 (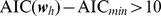), there is essentially no evidence that this model is useful.

### Software

We fitted the ERMM with the R package “Mixer” [24], [39]–[41]. The “mixer” function specifies default values for the maximum number of iterations, and we found improved performance by increasing these (nbiter = 80 up from 10, fpnbiter = 40 up from 5). We found 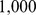 random restarts was sufficient to visit the optimal solution multiple times, but, to be exhaustive, we also considered up to 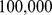 random restarts.

The Fast Louvain and Spectral algorithms were carried out using the Matlab “Brain Connectivity Toolbox” (http://www.brain-connectivity-toolbox.net/, accessed 15th June 2013) [49]. For the Fast Louvain algorithm, we used the function “modularity_louvain_und”, using 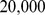
restarts. For the Spectral algorithm, we used the function “modularity_und”. This function is initiated on a randomly permuted adjacency matrix and although, in theory, all permutations of the adjacency matrix should provide the same result, some numerical discrepancies may occur during the spectral decomposition, subsequently leading to slightly different modularity fits. Specifically, the variability in the fits is driven by numerical errors in the estimation of the elements of 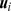, which can erroneously change the sign of its element. For example, if the true value of an element of 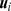 is 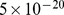 and the error is 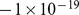, the estimated value would be 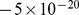. Indeed, this has an immediate impact on the vector 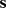 which will classify the corresponding node in the wrong group. To be exhaustive, we have therefore used 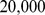 restarts.

To calculate the ARI scores, we used the function “adjustedRandIndex” in R software [58], [59] and, for the ICC and AIC, we use the R function “lmer” [60] that employs a Restricted Maximum Likelihood procedure [61] to obtain estimates of 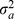, 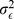 and AIC.

## Results

We first detail the estimated mesoscale structure of the *C. elegans* brain network found by the Erdős-Rényi Mixture Model, and then we proceed to compare these results with the ones obtained by the Fast Louvain and Spectral algorithms. The final part considers the generative properties of the Erdős-Rényi Mixture Model with respect to the network's degree distribution and clustering coefficient.

### Erdős-Rényi Mixture Model estimate of mesoscale structure in *C. elegans* brain network

**Figure 2:**
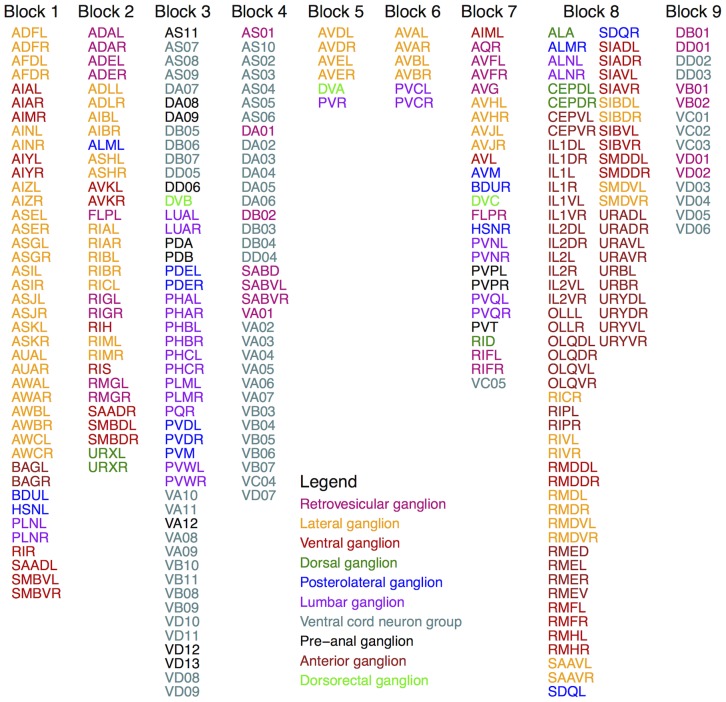
Classification of neurons for each Block of the ERMM fit. The corresponding neuron labels are colour coordinated according to their ganglion type.

**Figure 3:**
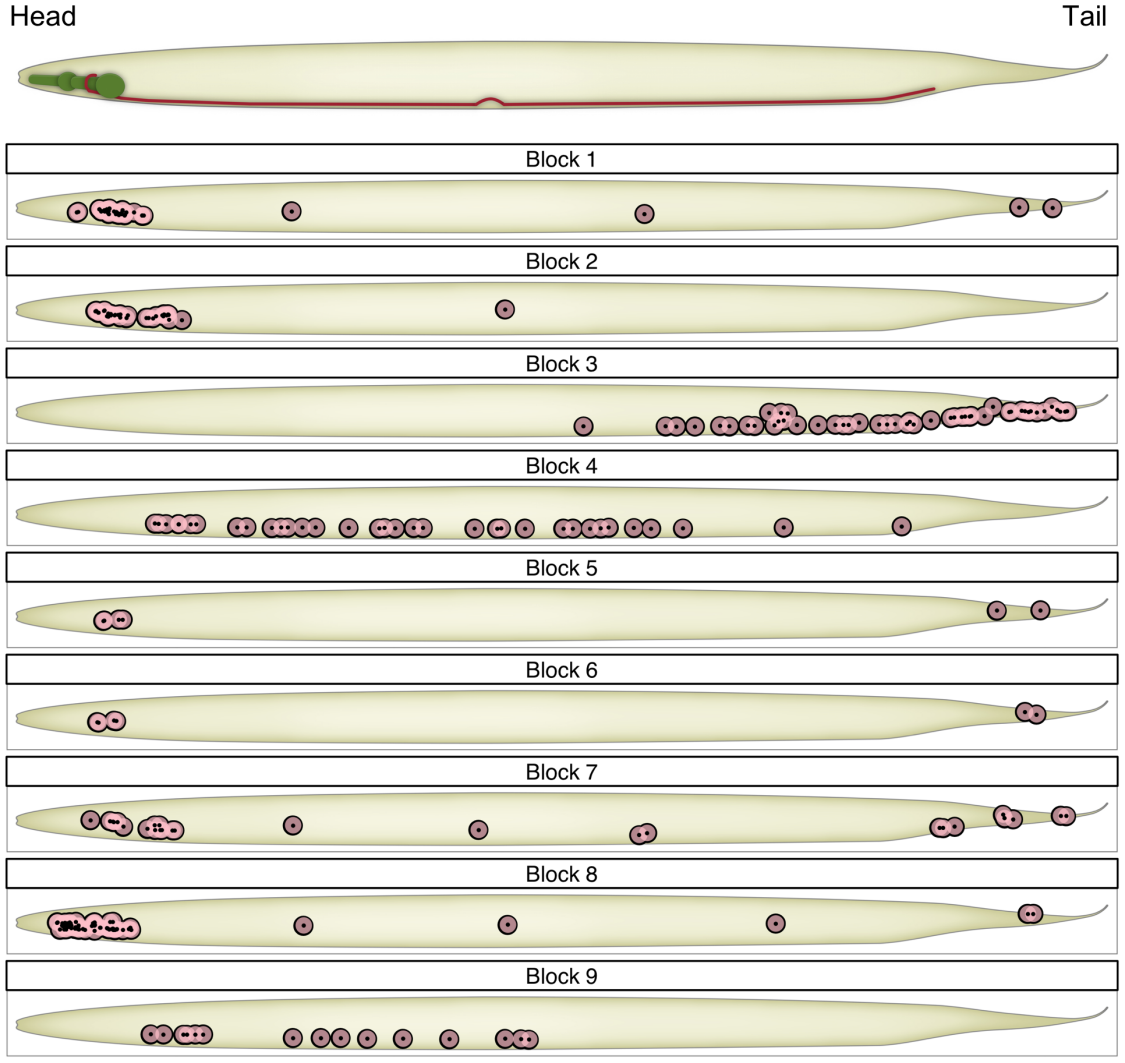
Anatomical locations of neurons (cell body) in the ERMM fit. Each Block is shown on an approximate template, obtained from http://www.wormatlas.org/, last accessed 9th October 2013.

The optimal Erdős-Rényi Mixture Model fit of the *C. elegans* brain network consists of 9 blocks, each of which is listed in Figure 2. In addition, the anatomical locations of neurons in each Block are given in Figure 3. Broadly speaking, we found that approximately 70% of the neurons in Block 1 are head sensory neurons involved in chemo/thermotaxis or chemo/thermosensation which modulate body movement. In contrast, most head sensory neurons (around 25%) in Block 2 are involved in more direct, reflex like and deterministic effects on body movement such as escape or avoidance behaviour, while almost 60% of the remaining neurons are ring interneurons (ADA, AIB, AVK, RIA, RIB, RIC, RIG, RIS, RMG, URX), about half of which have unknown function. Nevertheless, we characterised this block as “escape/avoidance” even though its function or perhaps functional homogeneity is not entirely clear. Next, more than half of neurons in Block 3 (55%) consists of mid-body and posterior ventral cord motor neurons, while almost all of the remaining neurons are posteriorly located sensory neurons (PDE, PHA, PHB, PHC, PLM, PVD, PVM), known to have quite a direct effect on motor neurons (e.g., PHA and PHB control extent of reversals in chemo-repulsion). We have labeled this group “motor (posterior)”, but we will revisit the possible causes for their inclusion. Similarly, close to 90% of neurons in Block 4 are made up of anteriorly located ventral cord motor neurons (AS, DA, DB, VA, VB, VD) which is therefore labeled as “motor (anterior)” group. The next two Blocks (5 & 6) are among the smallest in size, each with only 6 neurons. In particular, 4 neurons in Block 5 are command interneurons for (backward) locomotion (AVD, AVE), while the remaining 2 neurons are DVA (mechanosensory integration) and PVR (unknown function); whereas all 6 neurons in Block 6 are locomotion command interneurons. Next, Block 7 is mostly (about 65%) composed of neurons with unknown function, however, as 15% of the neurons seem to be involved in egg-laying and defecation, we have labeled it as “unknown/egg-laying/defecation” group. The largest number of neurons is found in Block 8 which appears to be predominantly (about 60%) composed of head motor neurons and nose touch mechanoreceptors (mainly located in the head), as well as, a numerous ring motor neurons. Many of these neurons are involved in both local search behaviour (RIV, SMDD, SMDV) and avoidance or aversive head withdrawal (ALN, IL1D, IL1V, OLQD, OLQV, RMD). We labeled this block “nose-touch/head motor”, but it may be interesting to further investigate whether this block could be subdivided into more specialised subunits. The remaining Block 9 is composed entirely (100%) of anterior ventral cord motor neurons of class DB, DD, VB, VC and VD - as previously discussed, Block 4 contains the remaining of anterior ventral cord motor neurons of type AS, DA, DB, VA, VB, VD.

Overall, these observations indicate that the ERMM based partition highlights functionally meaningful features of the system's mesoscale organization.

**Figure 4:**
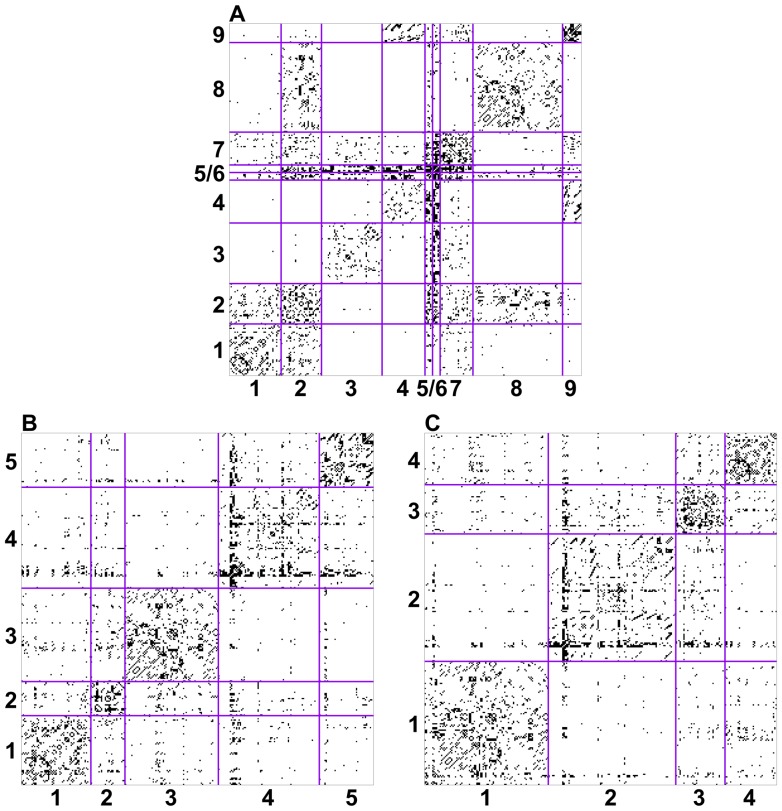
Reorganised adjacency matrices for each method. The groups are ordered arbitrarily; within each group, the neuron labels are sorted in alphabetical order. (**A**) The ERMM fit demonstrates the dense connections between - as well as within - certain blocks. This is in stark contrast to traditional community detection methods (**B**) and (**C**), that seek to find modules with dense intra-modular connectivity and sparse connections between modules. In addition, the ERMM fit defines blocks according to their internal and external connections. Thus, although Block 1 and 2 have similar within block connections, they are split because of their different connectivity with Block 8.

In Figure 4 (a), we show the optimal ERMM fit as a reorganised adjacency matrix. Note that the ERMM fit demonstrates the dense connections between - as well as within - certain groups. This is in stark contrast to traditional community detection methods that seek to find modules with dense intra-modular connectivity and sparse connections between modules. Instead, the ERMM classifies neurons into separate groups according to their individual connectivity profile to other groups, regardless of where connectivity happens to be dense. For example, Block 6 (AVA, AVB, PVC) comprises neurons with maximal interconnections (i.e., clique), which are, however, also fairly densely connected to the rest of the network. Note that Blocks 5 (AVE, AVD) and 6 (AVA, AVB, PVC) are separate groups because of differing internal connection rates (i.e., 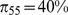 vs. 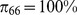) and external connection rates (e.g., 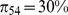 vs. 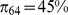). Also, consider Blocks 1 (chemo/thermosensation or chemo/thermotaxsis) and 2 (escape/avoidance) whose rates of internal and external block connections seem very similar, until it is noted that Block 1 has virtually no connections with Block 8 (nose touch mechanoreceptors and head motor neurons), while Block 2 is densely connected to Block 8.

**Figure 5:**
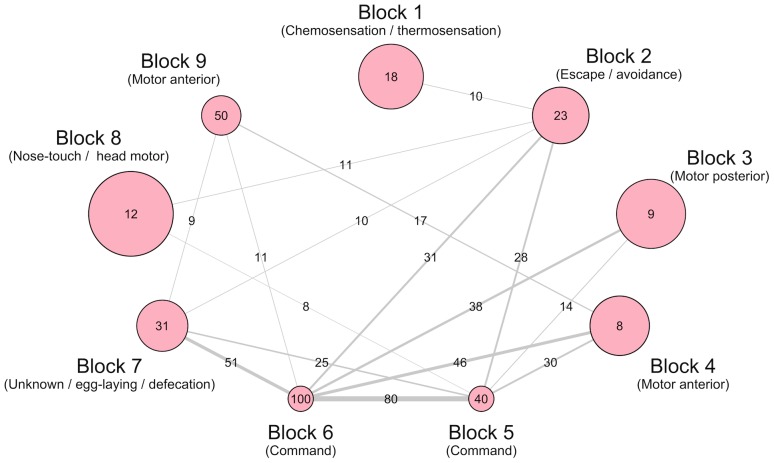
Compressed view of the *C. elegans* network, in terms of between/within block connection probability rates of the ERMM fit. The relative size of each circle indicates the number of neurons in that Block. The number inside the circle is the within-block connection probability in percent. The relative thickness of each line indicates the between-group connection strength, while the number on the edge gives the connection probability in percent (those less than 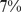 are omitted). Each Block is broadly characterised by its most representative function. Note how Blocks 1, 2 and 9 are “modules” with internal connectivity that is greater than external connectivity, while other structures are characterised by strong inter-block connectivity (e.g., Blocks 5 & 6 and Blocks 6 & 7). In addition, Block 6 (command interneurons) maintains relatively strong ties with the Block 3 and 4 (motor neurons) whose internal connections, however, are sparse, an example of core-periphery.

A concomitant advantage of the ERMM approach to community estimation is its ability to provide a compressed view of the original *C. elegans* network. As shown in Figure 5, this compressed view serves as a summary of the network's mesoscopic structure and reveals diverse patterns of connectivity between the blocks. Here, some blocks, such as Blocks 1, 6 and 8, appear to fit the standard definition of a “module” with high internal connectivity and sparse external connectivity. However, other structures which are characterised by strong communications between blocks are also present in the network; for example, Blocks 5 & 6 and Blocks 6 & 7, which may suggest that these are involved in the same functional circuit. In particular, Block 6 (command interneurons) - previously identified as a clique - maintains relatively strong ties with the Block 3 and 4 (motor neurons) whose internal connections, however, are sparse. This structure is known as the core-periphery and has been shown to be a functionally significant organisational structure in various real-world networks such as social networks or power grids [25], [62].

**Figure 6:**
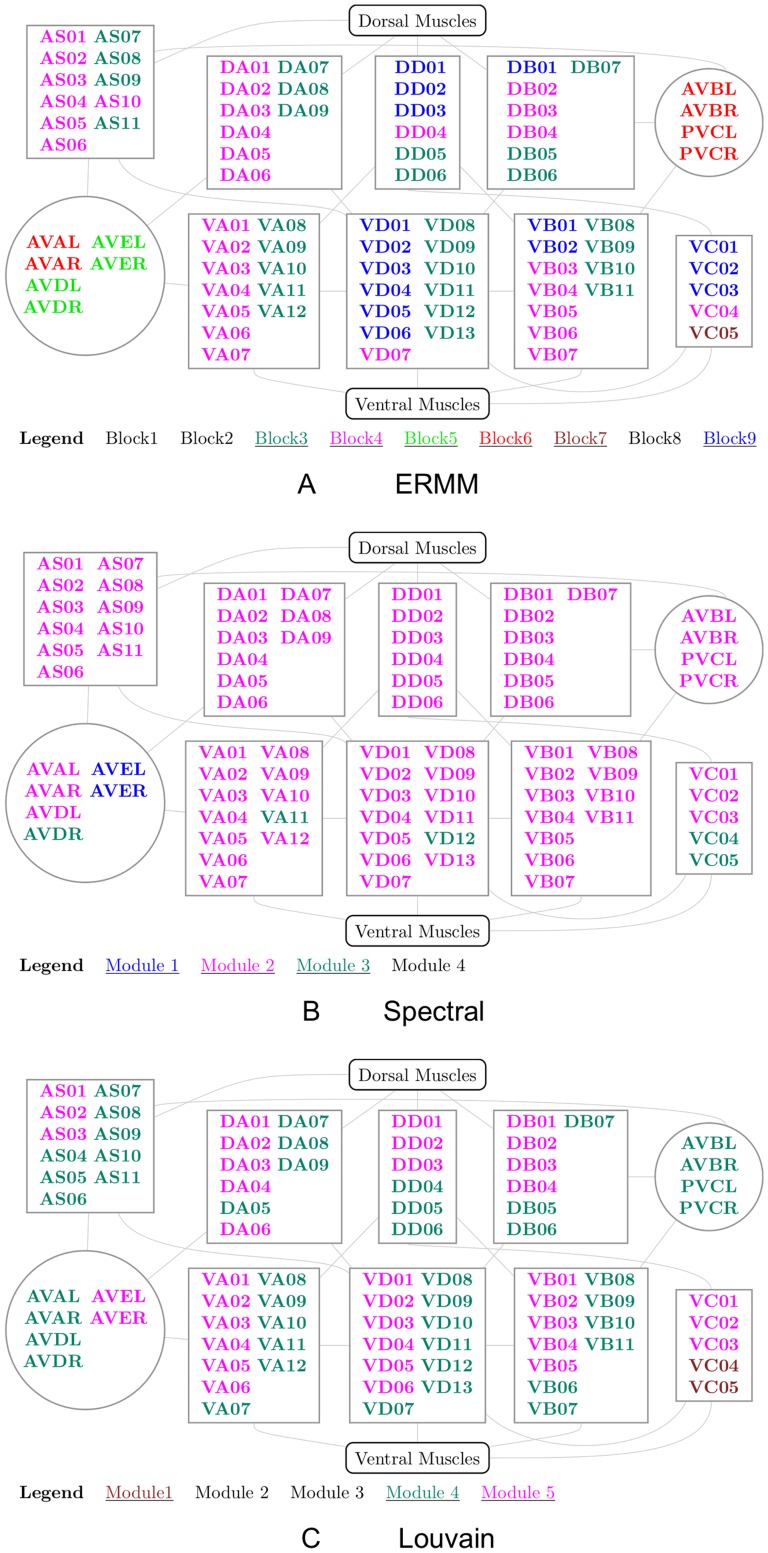
*C. elegans* locomotion network and estimated community structure for each method. Each subfigure shows the 74 Motor neurons (shown in rectangles) that support the animal's motion, which are divided into eight distinct groups. Four of these groups are connected to the ventral muscles (neuron labels: VA, VD, VB and VC; VC06 is omitted in our analysis), while the remaining four groups are connected to the dorsal muscles (neuron labels starting AS, DA, DD and DB). The remaining neurons (command neurons; shown in circles) belong to the category of interneurons; some are primarily required for promoting forward movements (labels starting PVC and AVB), while others promote backward movements (labels starting AVA, AVD and AVE). The colour of each neuron indicates the group membership from a particular method's partition. The ERMM fit (**A**) isolates the command neurons in Blocks 5 & 6, and distinguishes the posterior (Block 3) from the more anterior motor neurons (Blocks 4 & 9).

For further qualitative evaluation of the ERMM community estimate, we use the Locomotion circuit, as described in the accompanying text of Table 1. Figure 6 (a) shows a simplified diagram of this circuit (originally presented by Haspel et al. [30]) with the neuron block membership indicated by colour. The ERMM isolated the command interneurons into Blocks 5 and 6; while Blocks 4, 3 and 9 are fairly uniformly spread over all motor neurons. The distinction between these three blocks of motor neurons appears to be, at least partially, anatomically motivated, with the neurons in Block 3 being more posterior while the neurons in Blocks 4 and 9 are mainly found in the mid-section and anterior parts of the animal. Another noteworthy point is that the neurons VC04 and VC05, both implicated in egg-laying, are assigned separately to Blocks 4 and 7. The principal justification of this separation can be traced back to the network data used in this analysis where, for example, VC04 maintains connections to locomotion neurons AVB and AVH, while VC05 does not and, moreover, VC05 maintains connections to egg-laying neurons AVFL, AVFR, HSNR and PVT (Block 7), while VC04 does not. Given such differences in connection profiles between these two neurons, it is not surprising that they are separated. Relating to this, it is also worth mentioning that our network data excludes neuromuscular connections to the vulval muscles, made by both VC04 and VC05 which are the primary reason why these neurons are implicated in egg-laying behaviour.

### Comparison of the Erdős-Rényi Mixture Model fit with estimates from the Fast Louvain and Spectral algorithms

The Spectral and Fast Louvain algorithms decompose the *C. elegans* network into 4 and 5 modules with the maximal modularity scores of 0.402 and 0.411, respectively (Eq. (13) and (11)), indicating that both algorithms detect a prominent modular structure. As shown in the adjacency matrices in Figure 4 (b) and (c), both the Spectral and Louvain algorithms produced partitions with strong within-group connections and relatively sparse inter-group connections, as expected by definition.

**Figure 7:**
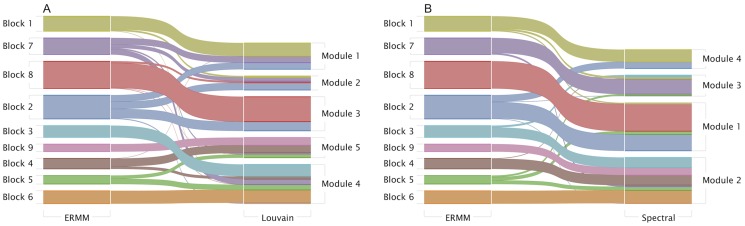
Correspondence between the ERMM fit and the estimates of Louvain (A) and Spectral (B) algorithms. The strands of the alluvial diagram show each block of the ERMM fit (on the left) and how these merge and split to form the modules of the Louvain and Spectral algorithms (on the right). The functional labels for the ERMM blocks are as follows. Block 1 (chemosensation/thermosensation), Block 7 (unknown/egg-laying/defecation), Block 8 (nose touch/head/motor), Block 2 (escape/avoidance), Block 3 (motor posterior), Block 9 (motor anterior), Block 4 (motor anterior), Block 5 (command) and Block 6 (command).

In order to compare the community structures obtained via all three methods, we plot an alluvial diagram (see Figure 7 (a) and (b)) showing each block of the ERMM method (on the left) and how these merge and split in order to make up the modules of the Louvain and Spectral partition. Strands of the alluvial diagram are coloured according to the block decomposition of the ERMM.

The first thing to note when observing this diagram is that the blocks obtained in the ERMM often roughly correspond to modules obtained via the other methods, with the Louvain and Spectral algorithm merging progressively more blocks into fewer modules. Secondly, we note that Blocks 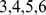 and 9 (mainly ventral cord motor neurons and interneurons controlling locomotion) are fairly well separated from Blocks 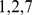 and 8 by all algorithms, so we will discuss these two subsets separately below.

Most nodes in Block 1 (chemo/thermo sensation) are also classed together in the other two algorithms, although they are also merged with some nodes from Blocks 2 (escape/avoidance) and 7 (mainly unknown function) in Module 1 of the Louvain algorithm. In contrast, the nodes in Block 2 are fairly dispersed in the Louvain algorithm (equally distributed between Modules 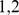 and 3), while they are split between Module 1 and 4 (mainly amphid neurons) by the Spectral algorithm. As noted earlier, neurons in Blocks 1 (chemo/thermo sensation) and 2 (escape/avoidance) are also tightly interconnected and they only fall as separate blocks because of their differential connectivity to nodes in Block 8 (nose-touch/head motor). The functional relevance of this finding is yet unclear but this pattern is biologically plausible and is a particularly striking aspect of the ERMM result (as shown in Figure 4 (a)). We also note that, while Block 8 seems to lump together many of the non-sensory neurons in the head, these neurons are also all grouped together by both the Spectral (Module 1) and the Louvain algorithm (Module 3). Nevertheless, it may be interesting to further investigate whether this block could be subdivided into more specialised subunits. One such approach could be to include in the analysis virtual nodes for the various external cues (chemical attractants, olfactory cues, temperature, touch, osmolarity, etc) or to include virtual nodes for the various muscle groups controlled by motor neurons, as this information has recently been shown to be useful in understanding the connectivity of motor neurons in the Locomotor system [63].

Looking at Block 7, we note that it corresponds quite well to Module 3 in the Spectral algorithm, but it is split between all modules (and mainly Modules 1 and 4) in the Louvain algorithm. From Figure 4 (a), it is clear that Block 7 has a very specific connectivity pattern. We therefore predict that this is likely to correspond to a biologically relevant functional grouping. This is particularly interesting because many of the neurons in this block have unknown functions and because these neurons are not anatomically co-located. Thus, in investigating the functional relevance of this block, it will be important to consider its particularly strong relationships to Blocks 2 and 6.

Now, turning our attention to Blocks 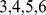 and 9, we note the following observations. Block 4 is made up almost entirely of anteriorly located ventral cord motor neurons (AS, DA, DB, VA, VB, VD), while Block 9 is composed entirely of anterior ventral cord motor neurons (DB, DD, VB, VC and VD). These two blocks are merged by both the Spectral and Louvain algorithms, and looking at Figure 4 (a), their separation into two different blocks does not seem to be a strong feature of the ERMM method either. It seems to be based by a differential connectivity to Block 7, but the effect is not very strong.

As previously mentioned, Block 3 is composed mostly of mid-body and posterior ventral cord motor neurons and almost all of the remaining neurons are posteriorly located sensory neurons (PDE, PHA, PHB, PHC, PLM, PVD, PVM). Almost all neurons in this block (including the posteriorly located sensory neurons listed) are also grouped together in Module 4 of the Louvain algorithm and almost all of them are in Module 2 of the Spectral algorithm. We however note that, in the Spectral (but not Louvain) partition, these neurons are also grouped together with the anterior ventral cord motor neurons of Blocks 4 and 9. While the roughly anatomical split between ventral cord motor neurons in the ERMM and Louvain method may not lead to new biological insights, it is certainly driven by a strong lack of connectivity between Blocks 4 (anterior) and 3 (posterior) which is a true feature of the data. It is worth noting that the connectivity data for *C. elegans* are known to be partial or missing for 39 of 302 neurons, including 21 of the 75 locomotor motoneurons [63] and the data for the posterior parts of the nerve cords are especially sparse and uncertain. It is therefore unclear whether this split between Blocks 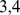 and 9 contains biological information or whether a more complete mapping of connections in the posterior part of the ventral cord would alter these results. Note, for example, that the split does not correspond to a division between neurons involved in forward and backward locomotion [30].

Finally, Blocks 5 and 6 are also merged with ventral cord motor neurons from Blocks 3 in both Louvain and Spectral algorithms. This is driven by the dense inter-connectivity between these nodes, however, the separation of Blocks 5 and 6 from the rest of the networks is one of the key features of the ERMM decomposition. Indeed, these blocks correspond almost exactly to the rich-club (core-periphery structure) whose functional importance has recently been confirmed [9].

It is also worth noting that compressed views of the network - see the ERMM fit in Figure 5 - are not available for the Fast Louvain and Spectral algorithms since these, by definition, decompose the network into modules with minimal connectivity between them.

Figure 6 (b) and (c) show the Locomotion circuit and the partitions found by the Spectral and Fast Louvain algorithms. In contrast to the ERMM model, both of these algorithms failed to distinguish the command neurons from the motorneurons. In the case of the Spectral algorithm, some of the command neurons like AVEL and AVER are isolated but the rest are mixed with the motorneurons. This effect may be explained by the rigid definition of the notion of “community” that is common to both algorithms. As we can observe, this particular *a priori* assumption does not allow the network's topology to dictate the form of the community structure, resulting in functionally less meaningful decompositions. Similar observations can be made about the neurons VC04 and VC05, which are merged by both algorithms despite their different connectivity profiles, inherent to the data. As we saw previously, these neurons are split in the ERMM partition.

Further quantifications of the solutions in terms of the separation of of L/R (left/right symmetric) neurons of the same class are presented in Table S1 in File S1. Here, we note that out of 92 L/R neuron pairs, contained in this data set, the ERMM and Spectral algorithm partitions assigned 85 such neuron pairs in the same groups and misclassified 7 pairs, while the Louvain partitions misclassified 5 pairs. In general, ALM and SAAD are separated by all methods, while other misclassified neurons appear to be distinct.

**Figure 8:**
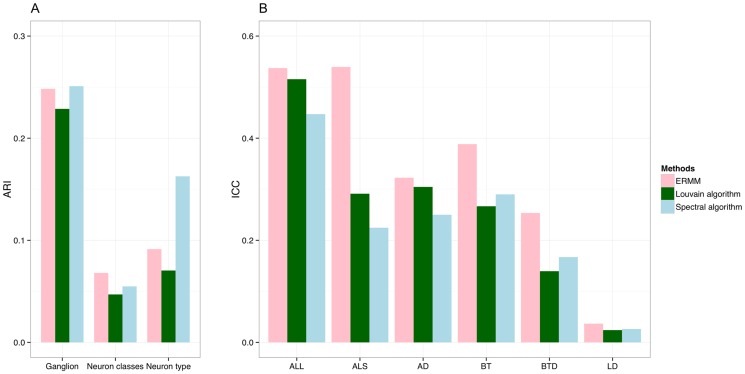
Method comparisons in terms of prior biological features. (**A**) ARI scores computed between three known biological classifications - Ganglion, Neuron classes (103 groups) and Neuron type (sensory, motor, interneuron and polymodal) - and the fits of each method. Collectively, the ARI scores are small and no greater than 0.26 for all 3 methods, suggesting that all 3 solutions are fairly different from the known biological partitions. (**B**) ICC scores for the Anatomical location (longitudinal) (ALL), Anatomical location (sectional) (ALS), Anatomical distance (AD), Birth time (BT), Birth time difference (BTD) and Lineage distance (LD). The ICC results indicate that the ERMM partition explains more biological variance than either of the other two methods. Compared to each other, ICC scores of Spectral and Louvain fits are largely similar.

Although the same block neurons in the ERMM partition appear to be functionally related, this overall partition does not correspond closely to the anatomical partition of neurons in 10 groups called ganglions (see Figure 2 and Figures S1 & S2 in File S1). More formal evaluations of this and other metrics, given in Figure 8 (a), use the ARI (Eq. (15)) scores to measure similarity between each of the known biological partitions (ganglion, neuron classes and neuron types) and each of the community estimate found by the methods. Collectively, the ARI scores are small and no greater than 0.26 for all 3 comparisons, with ganglion based partition being matched by the ERMM and Spectral algorithm with 0.25 ARI units, while the Louvain algorithm scored slightly lower. Compared to the 103 classes of neurons, the overall ERMM partition exhibits slightly higher ARI score than the partitions of the Louvain and Spectral algorithms, but note that these scores are still generally low. Similar observations hold for the ARI scores by neuron type, where the Spectral community estimate seems to be slightly more compatible than the other two fits, which tend to assign different neuron types to the same groups. These findings suggest that in general all 3 solutions are fairly different from the known biological partitions.

However, it has to be noted that although the ARI score can quantify the level of similarity between partitions of interest, it cannot infer (i.e., assign a P-value) on whether one partition has significantly better fit than another partition. This is statistically challenging as the solutions (fits) are sourced from the 3 different methods and, therefore, simple validation strategies like the one presented in Pan et al. [8] would not be appropriate. Nevertheless, using the ICC and AIC criterion, we can compare qualitatively all 3 estimates. For this, Figure 8 (b) shows the ICC (Eq. (17)) performance of the estimated partitions with respect to six quantitative biological features. The hypothesis here is that neurons that are implicated in the same function or behaviour might be similar in terms of these quantitative features, so biologically meaningful community structures should be composed of groups that are relatively homogeneous in terms of these metrics. We note that the six quantitative biological features were selected based on the datasets available, but not all are expected to be equally useful or revealing. For example, although one might expect lineage distance (LD) to be relevant, in fact neurons of the same class (typically involved in the same function) develop around the same time and usually have no immediate common precursors. This is reflected in low ICC scores in all three methods for lineage distance. Birth time (or BTD) is therefore expected to be more representative of function, and this is confirmed by higher ICC across all methods, with ERMM showing particularly good performance. Similarly, anatomical location (especially in the longitudinal direction) is expected to cluster functionally related neurons close together. This is because neuronal placement tends to minimise wiring [64] and neurons involved in the same function therefore benefit by being close together both for efficient inter-connections between these neurons and because they are likely to be receiving similar (localised) external cues or controlling similar (localised) muscle groups. Again, the ERMM shows superior ICC in all distance related metrics.

Overall, the ERMM partition provides the best ICC scores on all six biological features. For example, the ERMM partition explains over 50% of the variance in the sectional anatomical location (ALS), while the other two methods explain only about half as much variability. Also, we note that neither the Louvain or Spectral measures dominate one another on the basis of the ICC scores.

Finally, we use the AIC score (Eq. (18)) to assess if the differences between the partitions are significant. Table 2 shows the AIC score for each method and biological feature, and the minimum AIC score (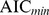) always corresponds to the ERMM fit. Using Eq. (19), we compute the relative likelihood to assess how much more likely it is that the Louvain or Spectral partition minimises the distance from the true partition versus the ERMM partition. As we can observe in Table 2, both the Louvain and Spectral fits fall far away from the bound 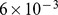
and, as such, they pose no evidence that these fits are more significant than the fit of the ERMM. In short, the AIC analysis unambiguously favours the ERMM fit as more compatible with the data, for all six of biological features, than the fits of the Louvain and Spectral algorithms.

**Table 2:** Individual AIC scores and relative likelihood of the ERMM, Spectral and Louvain partitions obtained on the set of biological features: Anatomical location (longitudinal) (ALL), Anatomical location (sectional) (ALS), Anatomical distance (AD), Birth time (BT), Birth time difference (BTD) and Lineage distance (LD).

Biological Feature	AIC scores			Relative Likelihood	
	ERMM	Louvain	Spectral	Louvain vs. ERMM	Spectral vs. ERMM
ALL	57.47	93.77	163.33	1.31×10^−8^	1.03×10^−23^
ALS	–1478.54	–1408.02	–1410.16	4.84×10^−16^	1.41×10^−15^
AD	12812.45	18913.31	26723.75	<1×10^−100^	<1×10^−100^
BT	4271.19	4300.52	4293.29	4.27×10^−7^	1.58×10^−5^
BTD	601475.22	605191.28	604905.85	<1×10^−100^	<1×10^−100^
LD	199676.54	200003.97	200136.19	7.94×10^−72^	1.54×10^−100^

The strength of evidence is computed to compare the Spectral and Louvain partitions against the ERMM.

### Erdős-Rényi Mixture Model and generative modelling of the *C. elegans* brain network

**Figure 9:**
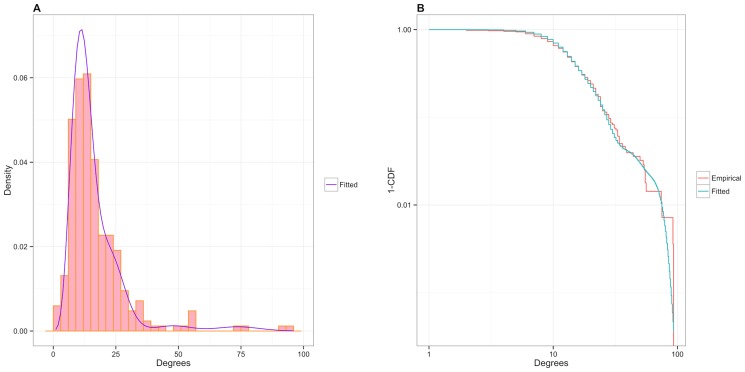
Observed and ERMM-based fit of the degree distribution. (**A**) Histogram of the empirical distribution with the ERMM fit. (**B**) Complementary cumulative distribution function (CDF) (i.e., 1-CDF) of the degrees and ERMM fit on the log-log scale. The ERMM-fitted distribution captures the large-scale features of the degree distribution extremely well, as well as most of the fine-scale features.

In addition to estimating the mesoscale structure, the ERMM also provides a generative model of the *C. elegans* nervous system which provides estimates of other network characteristics such as the empirical clustering coefficient (Eq. (1)) and degree distribution. Figure 9 shows the observed and fitted degree distribution, demonstrating that the ERMM provides a faithful approximation of the empirical degree distribution. The fit is based on a Poisson mixture (Eq. (9)), and Table 3 gives the estimated Poisson means (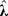
's) and weights (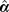
's). Notably, Block 6 (command neurons) has the greatest connectivity with an average degree of 74.23.

**Table 3:** Poisson mixture parameters 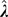 and mixture weights 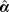 in the ERMM.

Block	1	2	3	4	5	6	7	8	9
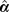	0.15	0.11	0.17	0.12	0.02	0.02	0.09	0.26	0.05
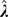	12.69	25.48	8.46	10.54	48.53	74.23	22.16	13.26	17.59

The model-based clustering coefficient from the ERMM is 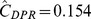, which is somewhat less than the empirical clustering coefficient 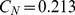. The likely explanation for this difference is that the ERMM specifies each edge as an independent Bernoulli trial (edges are formed with a given probability, independently from one another), which may underestimate the actual rate at which the triangles occur (two neurons connected to the same neighbour are also likely to connect to each other). To assess this, we conducted a small simulation, creating 100 adjacency matrices that followed the ERMM assumptions, using the *C. elegans* estimated parameters 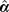 and 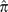 as truth. Based on these 100 realisations, the two clustering coefficients were quite similar, with 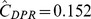 (SD 0.005) and 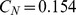 (SD 0.004), verifying that 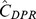 is a reasonable estimate of *C_N_* when the ERMM assumptions are satisfied.

### Practical considerations

Here, we report the computational times obtained on a 2.7 GHz quad-core Intel Core i7 linux host with 16 GB. The ERMM, on the default parameters setting (i.e., nbiter = 10, fpnbiter = 5) and the range 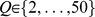, took 186 seconds, while the Louvain and Spectral algorithms took 0.07 and 0.62 seconds, respectively. Both the ERMM and Louvain methods required multiple restarts to find the optimal model, while restarts for the Spectral algorithm were needed due to numerical errors (see Section “Software” for more details). For the ERMM, on average, 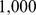 restarts were needed to visit the optimal model 12 times, while for the Louvain algorithm, over the total of 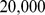 restarts, the optimal model was visited only once (for further details, see Figures S3 & S4 in File S1).

Also, to assess the stability of the ERMM solution across multiple restarts, we computed the ARI similarity score between the global optimal solution (see Table S2 in File S1), as well as the number of times that a solution was obtained out of 100,000 restarts. These results are consistent with the selected model (9 blocks) being the global optimum and, moreover, this solution occurs frequently over the restarts. Also, it is noteworthy to observe a considerable gap (of about 0.1 ARI units) between the optimal solution and the other solutions, which suggests that the optimal solution is well-identified and, furthermore, nearly optimal solutions are discernibly different. In the context of the stability of solutions, we also show the variability of ARI and ICC scores of each fit obtained from different restarts (Figure S5 in File S1).

## Discussion

Our results highlight the advantages in the use of the model-based Erdős-Rényi Mixture Model over the deterministic community detection algorithms. The mixture model decomposed the network into an interpretable set of 9 blocks, comprising 2 small blocks that correspond to the command interneurons, and 7 larger blocks that approximately correspond to the modules defined by the deterministic algorithms. Considering other work which points to the command interneurons of the *C. elegans* nervous system as the topological rich club [9], it seems that the ERMM decomposition has been able to capture both modular and core-periphery aspects [25], [65] of the mesoscale organization of the network. This conceptual scope, which can reconcile modular and core-periphery views of community structure, is a clear advantage of the ERMM compared to deterministic algorithms which are limited to an exclusive selection of one form over the other.

The block decomposition of the ERMM was also more successful at accounting for the prior biological data than either of the deterministic algorithms. Using the ICC metric to quantify the percentage of variance in a biological variable that is explained by any community structure, we found that the ERMM decomposition accounted for more than 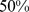 of the variance in anatomical location of the neurons, and more than 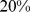 of the variance in anatomical distance of connections between neurons and neuronal birth times. Also, the ERMM explained a greater proportion of the variance in all biological variables than either of the deterministic algorithms.

Apart from considering the ICC scores, we subjected the fit of each method to a more rigorous procedure of model selection based on the AIC score. Computing the likelihood scores, we verified that the ERMM fit is significantly more compatible with the prior biological information than the fits of the Louvain and Spectral algorithms.

In addition to its advantages as an estimator, the ERMM also has technical advantages as a generative model (to simulate the network) and as a network compression basis to shrink the scale of a system. For example, we showed that the ERMM generated a good fit of the degree distribution and clustering coefficients of the *C. elegans* connectome. We also illustrated how the ERMM could be used to compress a graph into a set of super nodes, allowing a clearer view of the topology with fewer connections. In this sense, the ERMM provides a compression similar to power graph analysis methods [66], but it relaxes the condition for grouping nodes together, which allows for a more efficient and realistic compression.

We have found some shortcomings of the ERMM. For example, the mismatch between the empirical and model based clustering coefficient suggests that the stochastic model does not exactly match the data generating mechanism represented by the *C. elegans* nervous system. The ERMM can be extended by seeing it as a mixture Exponential Random Graph Model (ERGM) [67] where, conditional on the partitioning, the ERGM summary statistics are the edge counts in each of the 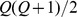 unique group pairs. Additional summary statistics can then be considered; for example, the triangle counts in each group pair. However, this will create a more complicated likelihood and necessitate new and yet more involved estimation procedures.

Nevertheless, the general practical advantage of the ERMM is that it leaves room for other distributional characterisations of edges which appear to be more in agreement with the network's specific type. Thus, for example, if this approach is used for the analysis of the weighted *C. elegans* network (i.e., the edge weights correspond to the total number of synaptic connections between a neuron pair), then the assumption that the edges are following a Binomial distribution is more appropriate. Furthermore, given that our study have used only a simple unweighted *C. elegans* network, it is interesting to compare our results to the 6 modules decomposition of the weighted *C. elegans* network reported by Pan et al. [8]. The corresponding extended results of this comparison (Figure S6 & Table S3 in File S1) show that the ERMM decomposition, again, explained more variance in the prior biological information, with the exception of the lineage distance where the ERMM explained 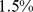 less than the 6 modules decomposition (Figure S6 in File S1). However, in terms of AIC score, the ERMM decomposition is uniformly more significant across all biological features than the decomposition reported by Pan et al. [8] (Table S3 in File S1).

Although the ERMM is classified as a stochastic blockmodel, it should not be confused with the *p*_1_ model that was proposed by Holland and Leinhardt [20]. In particular, the original *p*_1_ model does not aim to infer the connectivity rates of groups, which is why some authors like Wang and Wong [21] proposed different extensions. More recently, Karrer and Newman [68] considered the *p*_1_ model, referring to it as the standard stochastic blockmodel, in the context of undirected multi-graphs. They used heuristic arguments to derive a new model that corrects for variation in the degree distribution, named the degree corrected stochastic blockmodel. This approach treats node degree as a nuisance, to be discounted when finding groups. With our ERMM fit to *C. elegans*, this would not seem to be advantageous as it is the absolute differences in node degree that help define blocks. For example, consider Block 5 and 6 which have similar patterns of connections but their estimated connection rates are distinct (see Figure (5)). Furthermore, it is interesting to note that Karrer and Newman [68] found the standard stochastic blockmodel to be a poor fit to the Zachary karate club data [69], and their degree corrected model misclassified only one node. In contrast, we found that the ERMM reliably finds the 2 known groups in that data with zero errors.

Lastly, we found that the ERMM computational times are reasonable and depend on the range of blocks, the values of the internal parameters (nbiter, fpnbiter) and, also, the size of the network. It has been reported [24] that this approach can handle networks with several thousands of vertices, which is particularly impressive given the challenging likelihood optimisation. However, the problem of finding the global maximum is heavily dependent on the initialisation and, hence, we require restarts in order to carefully search the state space. While we used a cautious approach of running a large number of restarts (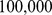), we found 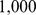 was sufficient to reliably identify the optimal model.

## Supporting Information

File S1 **Contains Figures S1–S6, Tables S1–S3, and Supplementary Text.**
(PDF)
