# Supplementary material for: Stochastic Blockmodeling of the Modules and Core of the Caenorhabditis elegans Connectome
Source: PLoS One. 2014 Jul 2;9(7):e97584. doi: 10.1371/journal.pone.0097584 (PMC4079667; doi:10.1371/journal.pone.0097584)
Supplement: File S1 — Contains Figures S1–S6, Tables S1–S3, and Supplementary Text. (PDF) [file pone.0097584.s001.pdf]

Supplementary Figures

Module 1			Module 2			Module 3	Module 4
ADEL	RIS	URYVL	AS01	PDER	VD05	AIML	ADAL
ADER	RIVL	URYVR	AS10	PHBL	VD06	AIMR	ADAR
ALA	RIVR		AS11	PHBR	VD07	ALML	ADFL
ALMR	RMDDL		AS02	PHCL	VD08	AQR	ADFR
ALNL	RMDDR		AS03	PHCR	VD09	ASJL	ADLL
ALNR	RMDL		AS04	PLML		ASJR	ADLR
AVEL	RMDR		AS05	PLMR		AVDR	AFDL
AVER	RMDVL		AS06	PVCL		AVFL	AFDR
AVKL	RMDVR		AS07	PVCR		AVFR	AIAL
AVKR	RMED		AS08	PVDL		AVG	AIAR
BAGL	RMEL		AS09	PVDR		AVHL	AIBL
BAGR	RMER		AVAL	PVM		AVHR	AIBR
CEPDL	RMEV		AVAR	PVR		AVJL	AINL
CEPDR	RMFL		AVBL	RID		AVJR	AINR
CEPVL	RMFR		AVBR	SABD		AVL	AIYL
CEPVR	RMGL		AVDL	SABVR		BDUL	AIYR
IL1DL	RMHL		AVM	VA01		BDUR	AIZL
IL1DR	RMHR		DA01	VA10		DVC	AIZR
IL1L	SAADR		DA02	VA12		HSNL	ASEL
IL1R	SAAVL		DA03	VA02		HSNR	ASER
IL1VL	SAAVR		DA04	VA03		PHAL	ASGL
IL1VR	SDQL		DA05	VA04		PHAR	ASGR
IL2DL	SDQR		DA06	VA05		PQR	ASHL
IL2DR	SIADL		DA07	VA06		PVNL	ASHR
IL2L	SIADR		DA08	VA07		PVNR	ASIL
IL2R	SIAVL		DA09	VA08		PVPL	ASIR
IL2VL	SIAVR		DB01	VA09		PVPR	ASKL
IL2VR	SIBDL		DB02	VB01		PVQL	ASKR
OLLL	SIBDR		DB03	VB10		PVQR	AUAL
OLLR	SIBVL		DB04	VB11		PVT	AUAR
OLQDL	SIBVR		DB05	VB02		PVWL	AWAL
OLQDR	SMBDL		DB06	VB03		PVWR	AWAR
OLQVL	SMBDR		DB07	VB04		RIFL	AWBL
OLQVR	SMBVR		DD01	VB05		RIFR	AWBR
PLNR	SMDDL		DD02	VB06		SABVL	AWCL
RIAL	SMDDR		DD03	VB07		VA11	AWCR
RIAR	SMDVL		DD04	VB08		VC04	PLNL
RIBL	SMDVR		DD05	VB09		VC05	RIR
RIBR	URADL		DD06	VC01		VD12	RMGR
RICL	URADR		DVA	VC02			SAADL
RICR	URAVL		DVB	VC03			SMBVL
RIGL	URAVR		FLPL	VD01			
RIGR	URBL		FLPR	VD10			
RIH	URBR		LUAL	VD11			
RIML	URXL		LUAR	VD13			
RIMR	URXR		PDA	VD02			
RIPL	URYDL		PDB	VD03			
RIPR	URYDR		PDEL	VD04			

Legend
Retrovesicular ganglion
Lateral ganglion
Ventral ganglion
Dorsal ganglion
Posterolateral ganglion
Lumbar ganglion
Ventral cord neuron group
Pre−anal ganglion
Anterior ganglion
Dorsorectal ganglion

Figure S1. Membership structure of the neurons in the Spectral fit. Neurons are coloured coded according to their ganglion type.

Module 1

ADAL, ADAR, ADFL, ADFR, ADLL, ADLR, AFDL, AFDR, AIAL, AIAR, AIBL, AIBR, AIML, AIMR, AINL, AINR, AIYL, AIYR, AIZL, AIZR, ASEL, ASER, ASGL, ASGR, ASHL, ASHR, ASIL, ASIR, ASJL, ASJR, ASKL, ASKR, AUAL, AUAR, AVFL, AVFR, AVHL, AVHR, AWAL, AWAR, AWBL, AWBR, AWCL, AWCR, HSNL, HSNR, PVQL, PVQR, RIFL, RIFR, RIR, RMGR, SMBVL, VC04, VC05

Module 2

ADER, ALNL, ALNR, AQR, AVKL, AVKR, BAGL, BAGR, DVC, PLNL, PLNR, PVPL, PVPR, PVT, RIGL, RIGR, RIML, RIMR, RMFL, RMFR, SAADL, SAADR, SAAVL, SAAVR, SMBDL, SMBDR, SMBVR

Module 3

ADEL, ALMR, CEPDL, CEPDR, CEPVL, CEPVR, IL1DL, IL1DR, IL1L, IL1R, IL1VL, IL1VR, IL2DL, IL2DR, IL2L, IL2R, IL2VL, IL2VR, OLLL, OLLR, OLQDL, OLQDR, OLQVL, OLQVR, RIAL, RIAR, RIBL, RIBR, RICL, RICR, RIH, RIPL, RIPR, RIS, RIVL, RIVR, RMDDL, RMDDR, RMDL, RMDR, RMDVL, RMDVR, RMED, RMEL, RMER, RMEV, RMGL, RMHL, RMHR, SDQR, SIADL, SIADR, SIAVL, SIAVR, SIBDL, SIBDR, SIBVL, SIBVR, SMDDL, SMDDR, SMDVL, SMDVR, URADL, URADR, URAVL, URAVR, URBL, URBR, URXL, URXR, URYDL, URYDR, URYVL, URYVR

Module 4

ALML, AS10, AS11, AS04, AS05, AS06, AS07, AS08, AS09, AVAL, AVAR, AVBL, AVBR, AVDL, AVDR, AVG, AVJL, AVJR, AVM, BDUL, BDUR, DA05, DA07, DA08, DA09, DB05, DB06, DB07, DD04, DD05, DD06, DVA, DVB, FLPL, FLPR, LUAL, LUAR, PDA, PDB, PDEL, PDER, PHAL, PHAR, PHBL, PHBR, PHCL, PHCR, PLML, PLMR, PQR, PVCL, PVCR, PVDL, PVDR, PVM, PVNL, PVNR, PVR, PVWL, PVWR, SDQL, VA10, VA11, VA12, VA07, VA08, VA09, VB10, VB11, VB06, VB07, VB08, VB09, VD10, VD11, VD12, VD13, VD07, VD08, VD09

Module 5

ALA, AS01, AS02, AS03, AVEL, AVER, AVL, DA01, DA02, DA03, DA04, DA06, DB01, DB02, DB03, DB04, DD01, DD02, DD03, RID, SABD, SABVL, SABVR, VA01, VA02, VA03, VA04, VA05, VA06, VB01, VB02, VB03, VB04, VB05, VC01, VC02, VC03, VD01, VD02, VD03, VD04, VD05, VD06

Legend

- Retrovesicular ganglion
- Lateral ganglion
- Ventral ganglion
- Dorsal ganglion
- Posterolateral ganglion
- Lumbar ganglion
- Ventral cord neuron group
- Pre−anal ganglion
- Anterior ganglion
- Dorsorectal ganglion

Figure S2. Membership structure of the neurons in the Louvain fit. Neurons are coloured to match their ganglion type.

ERMM				Spectral				Louvain			
AIMR	1	AIML	7	ALMR	1	ALML	3	RMGR	1	RMGL	3
BDUL	1	BDUR	7	RMGL	1	RMGR	4	SMBVL	1	SMBVR	2
HSNL	1	HSNR	7	PLNR	1	PLNL	4	ADER	2	ADEL	3
SADDL	1	SADDR	2	RMGL	1	RMGR	4	ALMR	3	ALML	4
ALML	2	ALMR	8	SAADR	1	SAADL	4	SDQR	3	SDQL	4
FLPL	2	FLPR	8	AVDL	2	AVDR	3				
RICL	2	RICR	8	SABVR	2	SABVL	3				

Table S1. Bilateral pairs of neurons split by each method. The neurons are listed according to their group assignment.

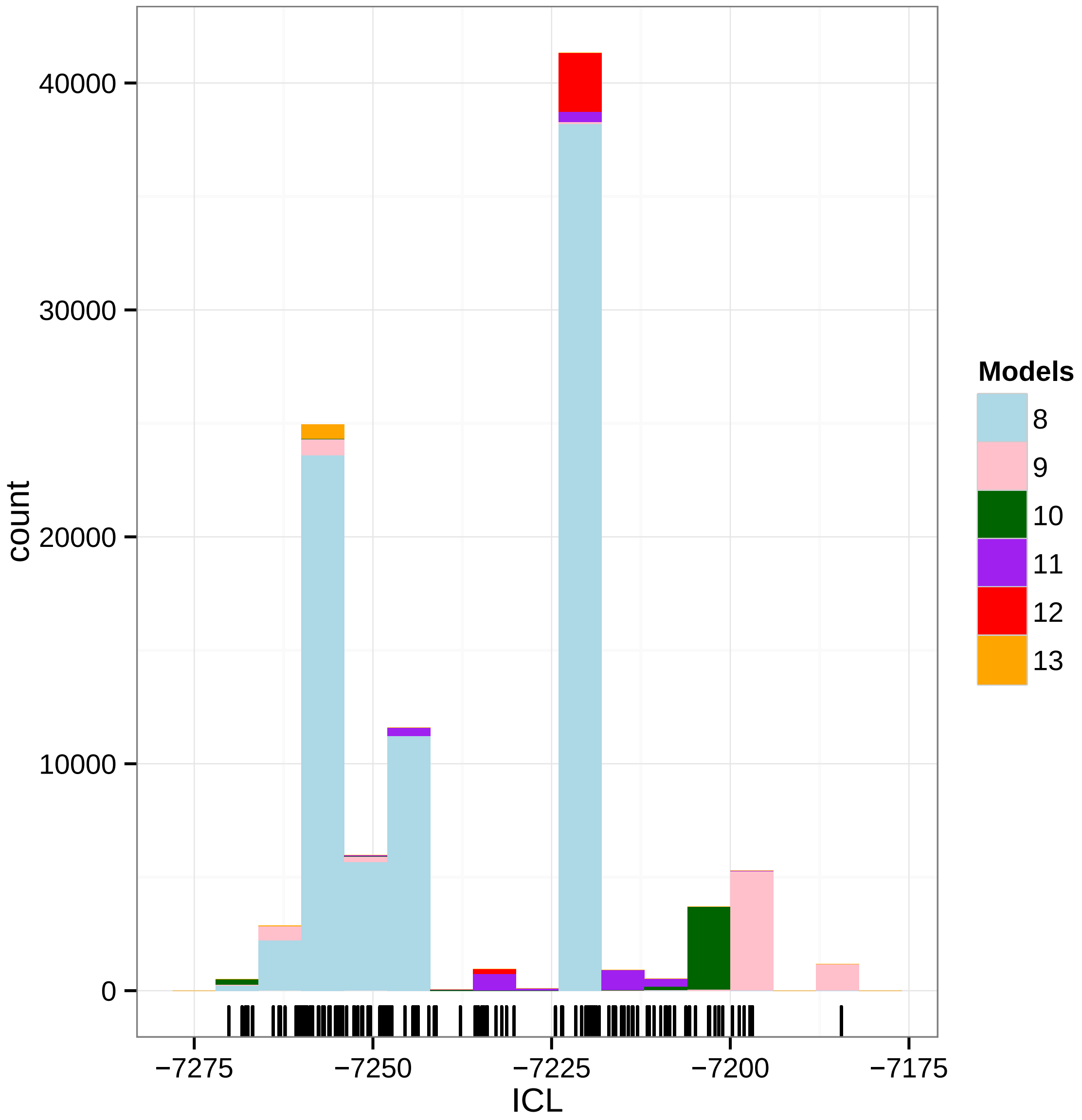

Figure S3. Model selection for ERMM. Histogram of the maximal ICL scores taken over 100, 000 restarts and given in terms of the number of classes in the corresponding fitted model. The number of classes within the models, that maximised the ICL scores, ranges from 8 to 13. The top ICL range (above −7218) is attained by the models with 9, 10 and 11 classes. Out of these, the model with 9 classes achieves the overall maximal ICL score of −7184.5 and this identical partition is repeated 1167 times.

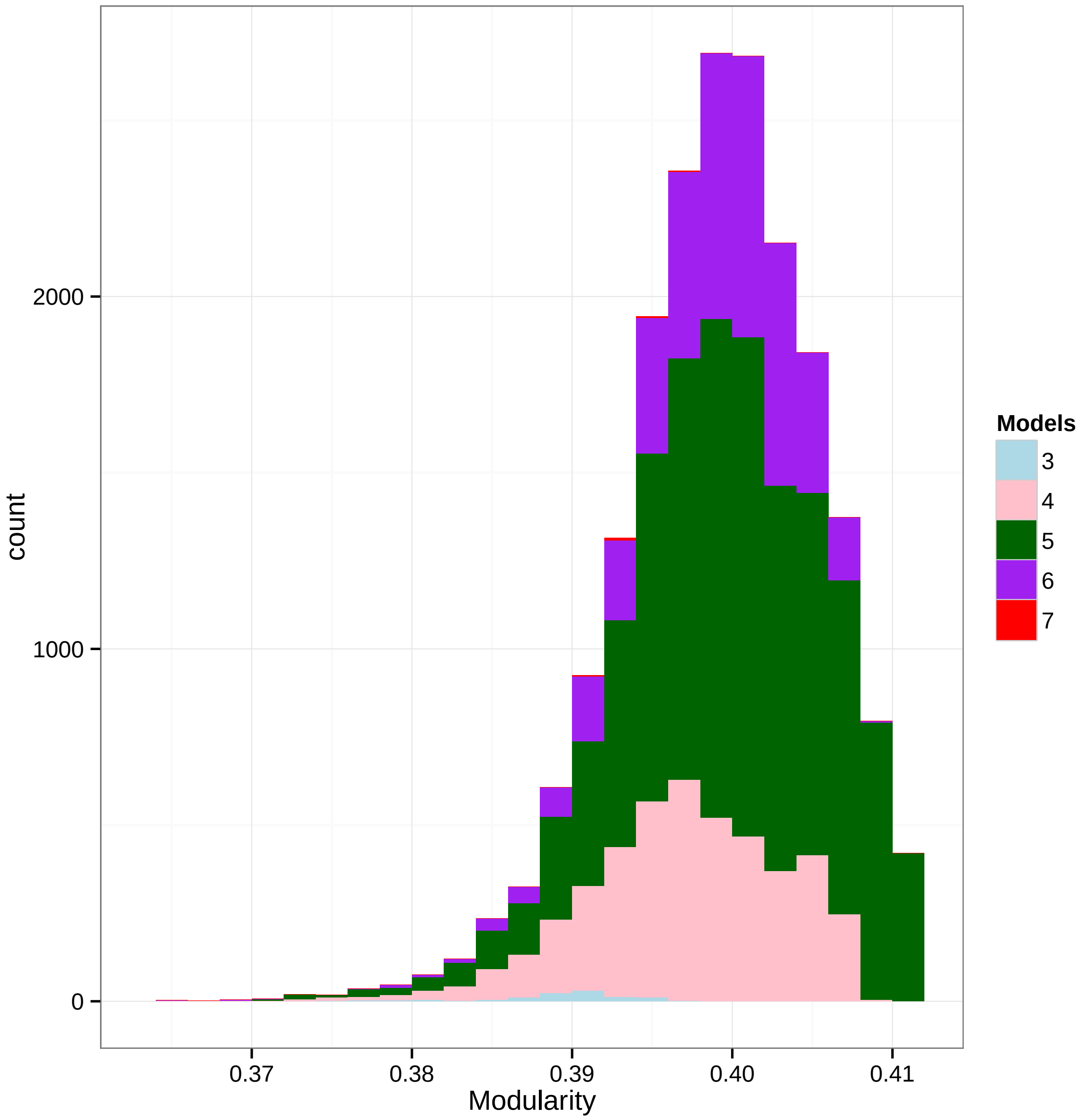

Figure S4. Model selection for Louvain algorithm. Histogram of modularity scores taken over $20,000$ restarts and given in terms of the number of classes in the corresponding model. The maximal modularity score (rounded to 3 decimals) 0.411 was achieved by the model with 5 classes and this was repeated 94 times. Note that the models with 5 classes are mutually different and that the best fit (i.e., the fit that achieves global maximum of the modularity score) occurs only once.

	ARI	Model	Frequency
1	1.00	9	1167
2	0.81	9	28
3	0.91	9	4827
4	0.75	11	31
5	0.79	9	8
6	0.91	9	82
7	0.92	9	319
8	0.89	9	39
9	0.61	10	28
10	0.90	10	183
11	0.89	9	9
12	0.92	10	1724
13	0.66	11	4
14	0.91	10	1256
15	0.89	10	163
16	0.89	10	301
17	0.92	10	24
18	0.69	9	18
19	0.71	11	52
20	0.72	11	123
21	0.69	10	73
22	0.83	9	7
23	0.62	11	177
24	0.79	9	14
25	0.70	10	46

Table S2. Evaluation of solution stability over restarts. The Adjusted Rand Index (ARI) expresses similarity between the optimal solution used in the paper and the 25 best solutions. There is a notable gap of about 0.1 ARI units between the optimal solution and solutions from other restarts of the algorithm. The frequency of each solution over the 100,000 restarts is also shown, as well as the corresponding number of groups in a fitted model.

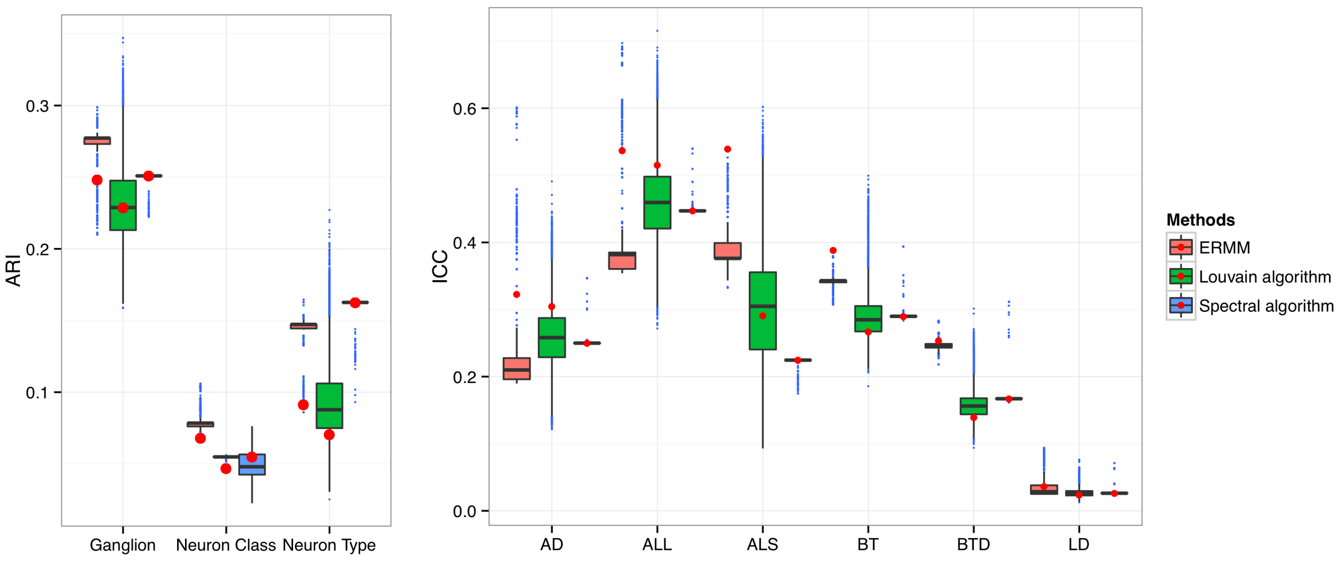

(a) **(b)**

Figure S5. Variation of ARI and ICC scores across restarts. The red points indicate the scores of the selected fits. In **(b)**, AD (Anatomical distance), ALL (Anatomical location longitudinal), ALS (Anatomical location sectional), BT (Birth time), BTD (Birth time difference), LD (Lineage distance).

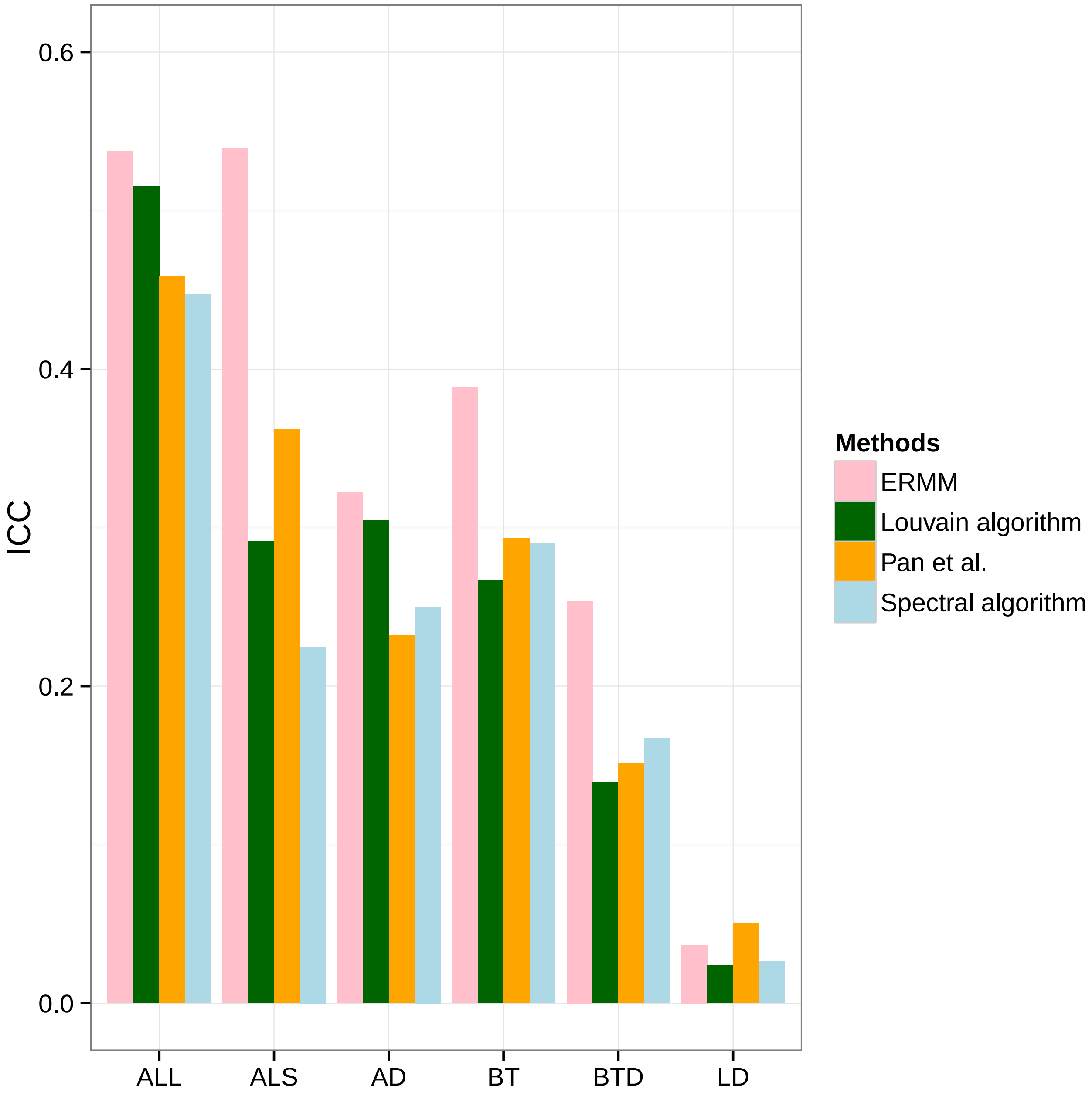

Figure S6. ICC scores of the partitions obtained with the ERMM, Louvain and Spectral algorithms on the unweighted *C. elegans* network and the 6 modules partition reported by Pan et al. [1], who used a modified Spectral algorithm to analyse a weighted *C. elegans* network. The ICC scores are measured over the range of biological features such as: ALL (Anatomical location longitudinal), ALS (Anatomical location sectional), AD (Anatomical distance), BT (Birth time), BTD (Birth time difference), LD (Lineage distance). The ERMM partition obtains the highest ICC scores, apart from the Lineage Distance, where the 6 module decomposition explained more variance. Also, it is interesting to observe the level of similarity between the results of the two Spectral algorithms, where the inclusion of the full weighted network seems to show a clear improvement of the ICC scores on the ALS and LD, but not on the other biological features.

Biological Feature	AIC scores			
	ERMM	Louvain	Spectral	Pan et al.
ALL	57.47	93.77	163.33	121.17
ALS	−1478.54	−1408.02	−1410.16	−1449.05
AD	12812.45	18913.31	26723.75	21631.78
BT	4271.19	4300.52	4293.29	4290.2360
BTD	601475.22	605191.28	604905.85	604085.08
LD	199676.54	200003.97	200136.19	199757.08

(a)

Biological Feature	Relative Likelihood		
	Louvain vs. ERMM	Spectral vs. ERMM	Pan et al. vs. ERMM
ALL	1.31×10^{-8}	1.03×10^{-23}	1.47×10^{-14}
ALS	4.84×10^{-16}	1.41×10^{-15}	3.94×10^{-7}
AD	$< 1\times10^{-100}$	$< 1\times10^{-100}$	$< 1\times10^{-100}$
BT	4.27×10^{-7}	1.58×10^{-5}	7.31×10^{-5}
BTD	$< 1\times10^{-100}$	$< 1\times10^{-100}$	$< 1\times10^{-100}$
LD	7.94×10^{-72}	1.54×10^{-100}	3.26×10^{-18}

(b)

Table S3. Assessing quality of fits. (a) Individual AIC scores of the ERMM, Spectral and Louvain partition, as well as the 6 modules partition reported by Pan et al. [1]. The AIC scores are computed on the set of biological features: Anatomical location (longitudinal) (ALL), Anatomical location (sectional) (ALS), Anatomical distance (AD), Birth time (BT), Birth time difference (BTD) and Lineage distance (LD). The lowest AIC score is uniformly maintained by the ERMM. **(b)** Comparison of each method and the method with the lowest AIC (i.e., the ERMM). In all cases, there is clearly no evidence that the the partitions of the Spectral, Louvain and 6 modules decomposition reported by Pan et al. [1] are more significant.

Supplementary Text

Erdős-Rényi Mixture Model

For comprehensiveness, we give a thorough review of the ERMM, proposed by Daudin, Picard and Robin [2], and we offer detailed and more complete proofs than found in the original references.

We define G to be a simple random graph which is fully specified by the binary and symmetric adjacency matrix $\boldsymbol{X} = ((X_{ij}))_{1\leq i,j\leq n}$. This matrix has several obvious characteristics, the first is that the principal diagonal is 0, since the graph is simple, and the second is that the number of data points in $\boldsymbol{X}$ is given by $n(n-1)/2$ which is just the count of entries in the upper or lower triangular matrix.

In order to describe the ERMM, we first concentrate on the assumptions about the nodes (vertices). For the graph G, the set of all vertices, labelled as $\{V_i\}_{i\in 1,\dots,n}$, is assumed to be divided into Q unknown

blocks where the membership structure of each such block is determined by a $1 \times Q$ dimensional vector $\boldsymbol{Z}_i = (Z_{i1}, \ldots, Z_{iQ})$, where $\boldsymbol{Z}$ denotes the set $\boldsymbol{Z} = \{\boldsymbol{Z}_i\}_{i \in 1,\ldots,n}$. In particular, the elements of $\boldsymbol{Z}_i$ are the mutualy independent latent variables Z_{iq} which label vertices according to their block membership, thus we have

$$Z_{iq} = \begin{cases} 0 \text{ when } V_i \notin q\text{-th block} \\ 1 \text{ when } V_i \in q\text{-th block.} \end{cases} \tag{1}$$

Furthermore, for a division of G into Q blocks, $\boldsymbol{Z}_i$ is assumed to follow the single trial multinomial (or categorical) distribution

$$\boldsymbol{Z}_i \sim \mathrm{M}(1; \boldsymbol{\alpha}) \tag{2}$$

where the parameter $\boldsymbol{\alpha}$ is the $Q \times 1$ vector of the probabilities $\boldsymbol{\alpha} = (\alpha_1, \ldots, \alpha_Q)$. Subsequently, the probability that a randomly chosen vertex in a network is located in a q-th block is given as

$$\mathrm{P}(Z_{iq} = 1) = \alpha_q, \tag{3}$$

with constraint that the $\sum_{q=1}^{Q} \alpha_q = 1$. The immediate interpretation of this assumption is that the vertex belongs to one group only and, in the common parlance, this is known as the hard partitioning. To complete description of the ERMM, we focus next on the assumptions about the edges. For this, the ERMM specifies that, given the block assignments of the vertices, the elements of $\boldsymbol{X}$ are conditionally independent Bernoulli random variables with rates given by their corresponding elements in the connectivity matrix $\boldsymbol{\pi} = ((\pi_{ql}))_{1 \le q,l \le Q}$. In other words, if a vertex V_i belongs to a block q and a vertex V_j belongs to block l, then

$$X_{ij} | Z_{iq} = 1, Z_{jl} = 1 \sim \text{Bernoulli } (\pi_{ql}), \tag{4}$$

or for the vertices V_i and V_j located in the same block

$$X_{ij} | Z_{iq} = 1, Z_{jq} = 1 \sim \text{Bernoulli } (\pi_{qq}), \tag{5}$$

For the subsequent proofs, it is convenient to express the elements of the connectivity matrix $\boldsymbol{\pi}$ as the

conditional probabilities

$$\pi_{ql} = \mathrm{P}(X_{ij} = 1 | Z_{iq} = 1, Z_{jl} = 1), \tag{6}$$

$$\pi_{qq} = \mathrm{P}(X_{ij} = 1 | Z_{iq} = 1, Z_{jq} = 1). \tag{7}$$

Following the traditional notation of Paul Erdős and Alfréd Rényi, we can define the Erdős-Rényi Mixture Model as $G = G(n, \boldsymbol{\pi})$ where, for a fixed Q, we have $Q(Q+1)/2$ mini Erdős-Rényi models (ERMs), posed not only on the blocks but, also, on the relationships between the blocks. Thus, just as in the ordinary ERM, we can obtain the distribution of degrees and this is summarised in the following proposition.

Proposition: In an Erdős-Rényi Mixture Model $G = G(n, \boldsymbol{\pi})$, given the class membership of a vertex, the conditional distribution of the degree of this vertex ($\rho(V_i)$) is Binomial (approximately Poisson)

$$\rho(V_i) | Z_{iq} = 1 \sim \mathrm{Bin}(n-1, \overline{\pi}_q) \approx \mathrm{Poi}(\lambda_q), \tag{8}$$

where $\overline{\pi}_q = \sum_{l=1}^{Q} \alpha_l \pi_{ql}$ and $\lambda_q = (n-1)\overline{\pi}_q$.

Proof We consider random variable $\rho(V_i) = \displaystyle\sum_{j=1}^{n} X_{ij}$. The value of this variable ($\rho(V_i)$) increases only when $X_{ij} = 1$ and because of this we need to consider the following probability:

$$\begin{aligned}
\mathrm{P}(X_{ij} = 1 | Z_{iq} = 1) &= \frac{\mathrm{P}(X_{ij} = 1, Z_{iq} = 1)}{\mathrm{P}(Z_{iq} = 1)} \\
&= \frac{\sum_{l=1}^{Q} \mathrm{P}(X_{ij} = 1, Z_{iq} = 1, Z_{jl} = 1)}{\mathrm{P}(Z_{iq} = 1)} \\
&= \frac{\sum_{l=1}^{Q} \mathrm{P}(X_{ij} = 1 | Z_{iq} = 1, Z_{lj} = 1)\mathrm{P}(Z_{iq} = 1, Z_{lj} = 1)}{\mathrm{P}(Z_{iq} = 1)} \\
&= \frac{\sum_{l=1}^{Q} \mathrm{P}(X_{ij} = 1 | Z_{iq} = 1, Z_{lj} = 1)\mathrm{P}(Z_{iq} = 1)\mathrm{P}(Z_{lj} = 1)}{\mathrm{P}(Z_{iq} = 1)} \\
&= \sum_{l=1}^{Q} \mathrm{P}(X_{ij} = 1 | Z_{iq} = 1, Z_{jl} = 1)\mathrm{P}(Z_{jl} = 1) \\
&= \sum_{l=1}^{Q} \pi_{ql}\alpha_l = \overline{\pi}_q.
\end{aligned}$$

Furthermore, $((X_{ij}))_{1 \leq i \neq j \leq n}$ are conditionally independent, given the classes of vertices V_i and V_j, al-

lowing us to conclude

$$\rho(V_i)|Z_{iq} = 1 \sim \text{Bin}(n-1, \overline{\pi}_q).$$

As the Binomial distribution can be approximated by the Poisson, we get

$$\rho(V_i)|\{Z_{iq} = 1\} \sim \text{Poi}(\lambda_q) \ \text{ where } \ \lambda_q = (n-1)\overline{\pi}_q.$$

With this, the distribution of degrees is then defined as a mixture of Poisson distributions such that

$$\text{P}(\rho(V_i) = k) = \sum_{q=1}^{Q} \text{P}(\rho(V_i) = k|Z_{iq} = 1)\text{P}(Z_{iq} = 1) = \sum_{q=1}^{Q} \alpha_q \frac{e^{-\lambda_q}\lambda_q^k}{k!}. \tag{9}$$

Maximising the likelihood with the variational approach

For a fixed Q and the parameters $\psi = \{\boldsymbol{\alpha}, \boldsymbol{\pi}\}$, the complete data log likelihood is given as

$$\log \mathcal{L}(\boldsymbol{x}, \boldsymbol{z}; \psi) = \sum_{i=1}^{n}\sum_{q=1}^{Q} z_{iq} \log\left[\alpha_q\right] + \frac{1}{2}\sum_{i\neq j}\sum_{q,l} z_{iq}z_{jl} \log\left[\pi_{ql}^{x_{ij}}(1-\pi_{ql})^{1-x_{ij}}\right]. \tag{10}$$

To verify this, we consider

$$\log \mathcal{L}(\boldsymbol{x}, \boldsymbol{z}; \psi) = \log \mathcal{L}(\boldsymbol{z}; \boldsymbol{\alpha}) + \log \mathcal{L}(\boldsymbol{x}|\boldsymbol{z}; \boldsymbol{\pi}).$$

As $\boldsymbol{Z}_i$ follows a multinomial distribution, its likelihood is given as

$$\mathcal{L}(\boldsymbol{z}; \boldsymbol{\alpha}) = \prod_{q=1}^{Q} \alpha_q^{n_q}, \ \text{ where } n_q = \sum_{i=1}^{n} z_{iq}.$$

Taking logarithms, we get

$$\log \mathcal{L}(\boldsymbol{z}; \boldsymbol{\alpha}) = \sum_{q=1}^{Q}\sum_{i=1}^{n} z_{iq} \log\left[\alpha_q\right].$$

Furthermore, we have

$$\mathcal{L}(\boldsymbol{x}|\boldsymbol{z}; \boldsymbol{\pi}) = \prod_{q,l}\prod_{i<j}\left[\pi_{ql}^{x_{ij}}(1-\pi_{ql})^{1-x_{ij}}\right]^{z_{iq}z_{jl}},$$

$$\log \ \mathcal{L}(\boldsymbol{x}|\boldsymbol{z}; \boldsymbol{\pi}) = \frac{1}{2}\sum_{i\neq j}\sum_{q,l} z_{iq}z_{jl} \log\left[\pi_{ql}^{x_{ij}}(1-\pi_{ql})^{1-x_{ij}}\right],$$

and combing everything completes the verification.

To estimate the model parameters, however, we need the likelihood of the observed data $\boldsymbol{X}$, which

is typically obtained by taking a sum over expression (10) with respect to all possible values of $\boldsymbol{Z}$. Unfortunately, this sum is not tractable and the standard strategy, like the Expectation Maximisation (EM) algorithm, provides some reduction in the computational burden but imposes a drastic reduction in the size of networks that can be handled by the analysis. To resolve these issues, Daudin, Picard and Robin [2] proposed to use the variational approach [3, 4] which requires that the distribution of $\boldsymbol{Z}$ is of the form

$$\mathrm{P}(\boldsymbol{Z} = \boldsymbol{z}; \boldsymbol{\tau}) = \prod_{i=1}^{n} P(\boldsymbol{Z}_i = \boldsymbol{z}_i; \boldsymbol{\tau}_i) = \prod_{i=1}^{n} \prod_{q}^{Q} \tau_{iq}^{z_{iq}}, \tag{11}$$

where $P(\boldsymbol{Z}_i = \boldsymbol{z}_i; \boldsymbol{\tau}_i)$ is the multinomial distribution with parameter $\boldsymbol{\tau}_i = (\tau_{i1}, \ldots, \tau_{iQ})$ and $\tau_{iq} = \mathrm{P}(Z_{iq} = 1|\boldsymbol{X} = \boldsymbol{x})$. The form of the joint distribution given in the expression (11) is suggested by the model assumption by which the latent variables are independent. In the context of the variational approximation, the goal is to maximise the following quantity

$$\mathcal{J}(\mathrm{P}(\boldsymbol{Z}); \psi, \boldsymbol{\tau}) = \log \mathcal{L}(\boldsymbol{x}; \psi) - \mathrm{KL}\big[\mathrm{P}(\boldsymbol{Z}; \boldsymbol{\tau}) || P(\boldsymbol{Z}|\boldsymbol{X} = \boldsymbol{x}; \boldsymbol{\tau}, \boldsymbol{\pi})\big], \tag{12}$$

where $KL[\cdot||\cdot]$ is the Kullback-Leibler divergence. This gives the following estimating equations.

Proposition: Given parameters $\boldsymbol{\alpha}$ and $\boldsymbol{\pi}$, the optimal variational parameters $\hat{\boldsymbol{\tau}}_i = \arg\max_{\{\boldsymbol{\tau}_i\}} \mathcal{J}(\mathrm{P}(\boldsymbol{Z}); \psi, \boldsymbol{\tau})$ satisfy the following point relation

$$\hat{\tau}_{iq} \propto \hat{\alpha}_q \prod_{i \neq j} \prod_{l} \left[\hat{\pi}_{ql}^{x_{ij}} (1 - \hat{\pi}_{ql})^{1 - x_{ij}} \right]^{\hat{\tau}_{jl}}. \tag{13}$$

Proof To show this $\mathcal{J}(\mathrm{P}(\boldsymbol{Z}); \psi, \boldsymbol{\tau})$ is maximised with respect to the variational parameter $\boldsymbol{\tau}_i$, subject to the constraint $\sum_{q=1}^{Q} \tau_{iq} = 1$, that is, the goal is to maximise the following quantity:

$$\mathcal{J}(\mathrm{P}(\boldsymbol{Z}); \psi, \boldsymbol{\tau}) + \sum_{i=1}^{n} \xi_i \left[\sum_{q=1}^{Q} \tau_{iq} - 1 \right], \tag{14}$$

where ξ_i is the Lagrange multiplier and $\mathcal{J}(\mathrm{P}(\boldsymbol{Z}); \psi, \boldsymbol{\tau})$ is given as

$$\mathcal{J}(\mathrm{P}(\boldsymbol{Z}); \psi, \boldsymbol{\tau}) = \sum_{i=1}^{n} \tau_{iq} \log[\alpha_q] + \frac{1}{2} \sum_{i \neq j} \sum_{q=1}^{Q} \sum_{l=1}^{Q} \tau_{iq} \tau_{jl} \log \left[\pi_{ql}^{x_{ij}} (1 - \pi_{ql})^{1 - x_{ij}} \right] - \sum_{i=1}^{n} \sum_{q=1}^{Q} \tau_{iq} \log[\tau_{iq}]. \tag{15}$$

Substituting for $\mathcal{J}(\mathrm{P}(\boldsymbol{Z});\psi,\boldsymbol{\tau})$ into equation (14), differentiating with respect to τ_{iq} and setting this expression to zero, we get

$$\begin{aligned}\log\left[\tau_{iq}\right] &= \log\left[\alpha_q\right] + \sum_{i\neq j}\sum_{l=1}^{Q}\tau_{jl}\log\left[\pi_{ql}^{x_{ij}}(1-\pi_{ql})^{1-x_{ij}}\right] + 1 + \xi_i \\ &= \log\left[\alpha_q\prod_{i\neq j}\prod_{l=1}^{Q}\left[\pi_{ql}^{x_{ij}}(1-\pi_{ql})^{1-x_{ij}}\right]^{\tau_{jl}}\exp\{1+\xi_i\}\right],\end{aligned} \tag{16}$$

allowing us to conclude

$$\hat{\tau}_{iq} \propto \hat{\alpha}_q\prod_{i\neq j}\prod_{l=1}^{Q}\left[\hat{\pi}_{ql}^{x_{ij}}(1-\hat{\pi}_{ql})^{1-x_{ij}}\right]^{\hat{\tau}_{jl}}. \tag{17}$$

Proposition: Given the variational parameters $\boldsymbol{\tau}_i$, the values of the parameters $\boldsymbol{\alpha}$ and $\boldsymbol{\pi}$ that maximise $\mathcal{J}(\mathrm{P}(\boldsymbol{Z});\psi)$ are

$$\hat{\alpha}_q = \frac{1}{n}\sum_{i=1}^{n}\hat{\tau}_{iq}, \quad \hat{\pi}_{ql} = \frac{\sum_{i\neq j}\hat{\tau}_{iq}\hat{\tau}_{jl}x_{ij}}{\sum_{i\neq j}\hat{\tau}_{iq}\hat{\tau}_{jl}}. \tag{18}$$

Proof Maximising with respect to $\boldsymbol{\alpha}$, subject to the constraint $\sum_{q=1}^{Q}\alpha_q = 1$

$$\mathcal{J}(\mathrm{P}(\boldsymbol{Z});\psi,\boldsymbol{\tau}) + \xi\left[\sum_{q=1}^{Q}\alpha_q\right], \tag{19}$$

this gives:

$$\hat{\alpha}_q = \frac{1}{n}\sum_{i=1}^{n}\hat{\tau}_{iq}. \tag{20}$$

Similarly, maximising with respect to $\boldsymbol{\pi}$

$$\hat{\pi}_{ql} = \frac{\sum_{i\neq j}\hat{\tau}_{iq}\hat{\tau}_{jl}x_{ij}}{\sum_{i\neq j}\hat{\tau}_{iq}\hat{\tau}_{jl}}. \tag{21}$$

Estimation of the number of blocks via the ICL criterion

The model selection is handled by the ICL criterion, which was proposed by Biernacki et al. [5]. The construction of the ICL criterion relays on the lemma which states that, if the prior parameter distributions

for a model with Q blocks $\mathcal{M}_Q$, $p(\boldsymbol{\alpha}|\mathcal{M}_Q)$ and $p(\boldsymbol{\pi}|\mathcal{M}_Q)$, are such that

$$p(\psi|\mathcal{M}_Q) = p(\boldsymbol{\alpha}|\mathcal{M}_Q)\ p(\boldsymbol{\pi}|\mathcal{M}_Q), \tag{22}$$

then

$$\log \mathcal{L}(\boldsymbol{x}, \boldsymbol{z}|\mathcal{M}_Q) = \log \mathcal{L}(\boldsymbol{x}|\boldsymbol{z}, \mathcal{M}_Q) + \log \mathcal{L}(\boldsymbol{z}|\mathcal{M}_Q). \tag{23}$$

Proposition: For a model $\mathcal{M}_Q$ with Q blocks, the ICL criterion is

$$ICL(\mathcal{M}_Q) = \max_{\psi} \log \big[\mathcal{L}(\boldsymbol{x}, \hat{\boldsymbol{z}}|\psi, \mathcal{M}_Q)\big] - \frac{1}{2}\frac{Q(Q+1)}{2} \log \big[\frac{n(n-1)}{2}\big] - \frac{Q-1}{2} \log \big[n\big], \tag{24}$$

where $\mathcal{M}_Q$ denotes a model with Q blocks and $\hat{\boldsymbol{z}}$ denotes its estimate such that the elements of $\hat{\boldsymbol{z}}_i$

$$\hat{z}_{iq} = \begin{cases} 1 & \text{if} \quad \arg\max_{\tau_{iq}} \{\hat{\boldsymbol{\tau}}_i\}, \\ 0 & \text{otherwise.} \end{cases} \tag{25}$$

Proof Considering,

$$\log \big[\mathcal{L}(\boldsymbol{x}, \boldsymbol{z}|\mathcal{M}_Q)\big] = \log \big[\mathcal{L}(\boldsymbol{x}|\boldsymbol{z}, \mathcal{M}_Q)\big] + \log \big[\mathcal{L}(\boldsymbol{z}|\mathcal{M}_Q)\big]. \tag{26}$$

The first term is obtained by application of large sample Laplace integral approximation (i.e., Bayesian Information Criterion BIC), so we have

$$\log \big[\mathcal{L}(\boldsymbol{x}|\boldsymbol{z}, \mathcal{M}_Q)\big] \simeq \max_{\boldsymbol{\pi}} \log \big[\mathcal{L}(\boldsymbol{x}|\boldsymbol{z}, \boldsymbol{\pi}, \mathcal{M}_Q)\big] - \frac{1}{2}\frac{Q(Q+1)}{2} \log \big[\frac{n(n-1)}{2}\big]. \tag{27}$$

For the second term we use the Dirichlet prior, $\mathcal{D}(\delta)$, as its conjugate is the multinomial distribution,

and we get

$$\log\big[\mathcal{L}(\boldsymbol{z}|\mathcal{M}_Q)\big] = \log\int \mathcal{L}(\boldsymbol{z}|\boldsymbol{\alpha},\mathcal{M}_Q)p(\boldsymbol{\alpha}|\mathcal{M}_Q)d\boldsymbol{\alpha},$$

$$= \log\int\prod_{q=1}^{Q}\alpha_q^{n_q}\frac{\Gamma(\sum_{q=1}^{Q}\delta)}{\prod_{q=1}^{Q}\Gamma(\delta)}\prod_{q=1}^{Q}\alpha_q^{\delta-1}d\boldsymbol{\alpha},$$

$$= \log\left[\frac{\Gamma(Q\delta)}{\Gamma(\delta)^Q}\right] + \log\frac{\prod_{q=1}^{Q}\Gamma(n_q+\delta)}{\Gamma(\sum_{q=1}^{Q}n_q+Q\delta)}\int\frac{\Gamma(\sum_{q=1}^{Q}n_q+Q\delta)}{\prod_{q=1}^{Q}\Gamma(n_q+\delta)}\prod_{q=1}^{Q}\alpha_q^{n_q+\delta-1}d\boldsymbol{\alpha},$$

$$= \log\big[\Gamma(Q\delta)\big] - Q\log\big[\Gamma(\delta)\big] + \sum_{q=1}^{Q}\log\big[\Gamma(n_q+\delta)\big] - \log\big[\Gamma(n+Q\delta)\big].$$

Setting $\delta = \frac{1}{2}$, as it corresponds to the Jeffreys prior, and replacing $\boldsymbol{z}$ with its estimate $\hat{\boldsymbol{z}}$, we get:

$$= \log\big[\Gamma(Q\frac{1}{2})\big] - Q\log\big[\Gamma(\frac{1}{2})\big] + \sum_{q=1}^{Q}\log\big[\Gamma(\hat{n}_q+\frac{1}{2})\big] - \log\big[\Gamma(n+Q\frac{1}{2})\big].$$

Taking the expression for large n and approximating the Gamma function with Stirling's formula

$$\log\big[\mathcal{L}(\hat{\boldsymbol{z}}|\mathcal{M}_Q)\big] \simeq \sum_{q=1}^{Q}\hat{n}_q\log[\hat{n}_q] - n\log[n] - \frac{Q-1}{2}\log[n],$$

$$\log\big[\mathcal{L}(\hat{\boldsymbol{z}}|\mathcal{M}_Q)\big] \simeq \max_{\alpha}\log\big[\mathcal{L}(\hat{\boldsymbol{z}}|\alpha,\mathcal{M}_Q) - \frac{Q-1}{2}\log[n].$$

Finally, we get: $\log\big[\mathcal{L}(\boldsymbol{x},\boldsymbol{z}|\mathcal{M}_Q)\big] \approx \max_{\psi}\log\big[\mathcal{L}(\boldsymbol{x},\hat{\boldsymbol{z}}|\psi,\mathcal{M}_Q)\big] - \frac{1}{2}\frac{Q(Q+1)}{2}\log\big[\frac{n(n-1)}{2}\big] - \frac{Q-1}{2}\log\big[n\big] = ICL(\mathcal{M}_Q)$.

The Clustering Coefficient

The probabilistic definition of the clustering coefficient as proposed by Daudin, Picard and Robin in [2] is given as follows.

Proposition: In the ERMM, the clustering coefficient is

$$\hat{C}_{\mathrm{DPR}} = \frac{\sum_{q,l,s}\hat{\alpha}_q\hat{\alpha}_l\hat{\alpha}_s\hat{\pi}_{ql}\hat{\pi}_{qs}\hat{\pi}_{ls}}{\sum_{q,l,s}\hat{\alpha}_q\hat{\alpha}_l\hat{\alpha}_s\hat{\pi}_{ql}\hat{\pi}_{qs}}. \tag{28}$$

Proof

$$C_{\text{DPR}} = \text{P}(X_{ij}X_{jk}X_{ik} = 1 | X_{ij}X_{ik} = 1) = \frac{\text{P}(X_{ij}X_{jk}X_{ik} = 1)}{\text{P}(X_{ij}X_{ik} = 1)},$$
$$= \frac{\sum_{q,l,s} \text{P}(X_{ij}X_{jk}X_{ik} = 1, Z_{iq} = 1, Z_{jl} = 1, Z_{ks} = 1)}{\sum_{q,l,s} \text{P}(X_{ij}X_{ik} = 1, Z_{iq} = 1, Z_{jl} = 1, Z_{ks} = 1)},$$
$$= \frac{\sum_{q,l,s} \text{P}(X_{ij} = 1, X_{jk} = 1, X_{ik} = 1, Z_{iq} = 1, Z_{jl} = 1, Z_{ks} = 1)}{\sum_{q,l,s} \text{P}(X_{ij} = 1, X_{ik} = 1, Z_{iq} = 1, Z_{jl} = 1, Z_{ks} = 1)},$$
$$= \frac{\sum_{q,l,s} \hat{\alpha}_q \hat{\alpha}_l \hat{\alpha}_s \hat{\pi}_{ql} \hat{\pi}_{qs} \hat{\pi}_{ls}}{\sum_{q,l,s} \hat{\alpha}_q \hat{\alpha}_l \hat{\alpha}_s \hat{\pi}_{ql} \hat{\pi}_{qs}} = \hat{C}_{\text{DPR}},$$

where for the last step, we used conditional independence of $\boldsymbol{X}$ and the independence of $\boldsymbol{Z}$.

Spectral Algorithm

The problem of community detection is usually defined as finding the partition of a network into communities of densely connected vertices while minimising the number of connections between the communities. The goodness of a graph partition is generally assessed with a *quality* function whose the most frequently used version is known as *modularity* and it was proposed by Newman and Girvan [6]. The idea behind the concept of modularity is that the communities are found by the comparison of the actual density of connections in the subgraphs and the density one would expect to find if the vertices were connected at random. This random version of the original graph, that acts as a reference point in the modularity function, is called a *null model* and, typically, it is tailored to preserve some of the features of the original graph like the same number of edges or the same degree distribution [7–9].

In practice, the standard null model of modularity preserves the degree distribution of the original graph by generating half-edges so that each vertex in a null model receives as many half-edges as its corresponding degree in the original graph. Thus, the probability of randomly picking V_i is expressed as a proportion of V_i's degree in the total sum of the degrees, that is, $\rho(V_i)/2m$. Furthermore, the probability that vertices V_i and V_j form a complete edge is given as: $\rho(V_i)\rho(V_j)/4m^2$, while the expected count of

edges considered for V_i and V_j is $\rho(V_i)\rho(V_j)/2m := P_{ij}$. According to this, the modularity is defined as

$$\begin{aligned} f_{mod} &= \frac{1}{2m}\sum_{i,j}\Big(A_{ij} - P_{ij}\Big)\delta(c_i, c_j) \\ &= \frac{1}{2m}\sum_{i,j}\Big(A_{ij} - \frac{\rho(V_i)\rho(V_j)}{2m}\Big)\delta(c_i, c_j), \end{aligned} \tag{29}$$

where c_i and c_j denote the communities of vertices V_i and V_j, respectively, while $\delta(c_i, c_j) = 1$ if V_i and V_j are located in the same community, 0 otherwise.

The Spectral algorithm [10, 11] optimises the modularity by utilising the eigenvalues and eigenvectors associated with the modularity matrix $\boldsymbol{D}$, whose elements are

$$D_{ij} = A_{ij} - \frac{\rho(V_i)\rho(V_j)}{2m}. \tag{30}$$

Let $\boldsymbol{s}$ be an indicator vector which decomposes the nodes into 2 communities, with $s_i = 1$ if the vertex V_i is located in the first community and $s_i = -1$ if the vertex is located in the second community. This modifies the modularity function (29) as follows

$$\begin{aligned} f_{mod} &= \frac{1}{4m}\sum_{i,j}\Big(A_{ij} - \frac{\rho(V_i)\rho(V_j)}{2m}\Big)(s_i s_j + 1) \\ &= \frac{1}{4m}\sum_{i,j} D_{ij}s_i s_j + \frac{1}{4m}\sum_{i}\big[\sum_{j} A_{ij} - \frac{\rho(V_i)}{2m}\sum_{j}\rho(V_j)\big] \\ &= \frac{1}{4m}\sum_{i,j} D_{ij}s_i s_j + \frac{1}{4m}\sum_{i}\big[\rho(V_i) - \frac{\rho(V_i)}{2m}2m\big] \\ &= \frac{1}{4m}\sum_{i,j} D_{ij}s_i s_j \\ &= \frac{1}{4m}\boldsymbol{s}^T\boldsymbol{D}\boldsymbol{s}. \end{aligned} \tag{31}$$

Moreover, the vector $\boldsymbol{s}$ can be written as a linear combination of the normalised eigenvectors $\boldsymbol{u}_i$ associated with the matrix $\boldsymbol{D}$, thus, $\boldsymbol{s} = \sum_i a_i\boldsymbol{u}_i$ and $a_i = \boldsymbol{u}_i^T\boldsymbol{s}$. Using this along with the fact that β_i is the

eigenvalue of $\boldsymbol{D}$ corresponding to the eigenvector $\boldsymbol{u}_i$, we get

$$\begin{aligned}
\mathcal{Q}_{mod} &= \frac{1}{4m}\Big(\sum_i a_i \boldsymbol{u}_i^T\Big)\boldsymbol{D}\Big(\sum_j a_j \boldsymbol{u}_j\Big) \\
&= \frac{1}{4m}\sum_i a_i \sum_j a_j \boldsymbol{u}_i^T \boldsymbol{D}\boldsymbol{u}_j \quad \text{using} \;\; \boldsymbol{u}_i^T\boldsymbol{D} = \beta_i \boldsymbol{u}_i^T \\
&= \frac{1}{4m}\sum_i a_i \sum_j a_j \beta_i \boldsymbol{u}_i^T \boldsymbol{u}_j \quad \text{this is non-zero when} \;\; i = j \\
&= \frac{1}{4m}\sum_i a_i^2 \beta_i \\
&= \frac{1}{4m}\sum_i \big(\boldsymbol{u}_i^T \boldsymbol{s}\big)^2 \beta_i. \qquad (32)
\end{aligned}$$

The idea is to look for the largest positive eigenvalue of $\boldsymbol{D}$, and then group the vertices according to the elements of the corresponding eigenvector.

The extension of the algorithm to more than two communities is reflected in the consideration of the additional contribution Δf_{mod} to the modularity after dividing a community g with size n_g into two communities

$$\begin{aligned}
\Delta f_{mod} &= \frac{1}{2m}\Bigg[\frac{1}{2}\sum_{i,j\in g} D_{ij}(s_i s_j + 1) - \sum_{i,j\in g} D_{i,j}\Bigg] \\
&= \frac{1}{2m}\Bigg[\frac{1}{2}\sum_{i,j\in g} D_{ij} s_i s_j - \frac{1}{2}\sum_{i,j\in g} D_{ij}\Bigg] \\
&= \frac{1}{4m}\Bigg[\sum_{i,j\in g} D_{i,j} s_i s_j - \sum_{i,j\in g} D_{ij}\Bigg] \\
&= \frac{1}{4m}\sum_{i,j\in g}\Bigg[D_{ij} - \delta_{ij}\sum_{k\in g} D_{ik}\Bigg] s_i s_j \\
&= \frac{1}{4m}\boldsymbol{s}^T \boldsymbol{D}^{(g)} \boldsymbol{s}, \qquad (33)
\end{aligned}$$

where for the last step, we note that using the Kronecker delta notation δ_{ij}, we can write

$$\sum_{i,j\in g} D_{ij} = \sum_{i\in g}\delta_{ii}\sum_{j\in g} D_{ij} s_i^2 = \sum_{i,j\in g}\delta_{ij}\sum_{k\in g} D_{ik} s_i s_j,$$

such that $\boldsymbol{D}^{(g)}$ is $n_g \times n_g$ matrix whose elements are: $D_{ij}^{(g)} = D_{ij} - \delta_{ij}\sum_{k\in g} D_{ik}$.

The algorithm stops when there are no more positive eigenvalues.

References

1. Pan R, Chatterjee N, Sinha S (2010) Mesoscopic organization reveals the constraints governing *Caenorhabditis elegans* nervous system. PloS one 5: e9240.

2. Daudin J, Picard F, Robin S (2008) A mixture model for random graphs. Statistics and computing 18: 173–183.

3. Jordan M, Ghahramani Z, Jaakkola T, Saul L (1999) An introduction to variational methods for graphical models. Machine learning 37: 183–233.

4. Jaakkola T (2001) 10 tutorial on variational approximation methods. Advanced mean field methods: theory and practice : 129.

5. Biernacki C, Celeux G, Govaert G (2000) Assessing a mixture model for clustering with the integrated completed likelihood. Pattern Analysis and Machine Intelligence, IEEE Transactions on 22: 719–725.

6. Newman M, Girvan M (2004) Finding and evaluating community structure in networks. Physical review E 69: 026113.

7. Luczak T (1989) Sparse random graphs with a given degree sequence. In: Proceedings of the Symposium on Random Graphs, Poznan. pp. 165–182.

8. Molloy M, Reed B (1995) A critical point for random graphs with a given degree sequence. Random Structures & Algorithms 6: 161–180.

9. Pattison P, Robins G (2007). Handbook of probability theory with applications. chapter probabilistic network theory.

10. Newman M (2006) Modularity and community structure in networks. Proceedings of the National Academy of Sciences 103: 8577–8582.

11. Rubinov M, Sporns O (2010) Complex network measures of brain connectivity: uses and interpretations. Neuroimage 52: 1059–1069.
